# Transcriptomic analyses of gastrulation-stage mouse embryos with differential susceptibility to alcohol

**DOI:** 10.1242/dmm.049012

**Published:** 2021-06-17

**Authors:** Karen E. Boschen, Travis S. Ptacek, Matthew E. Berginski, Jeremy M. Simon, Scott E. Parnell

**Affiliations:** 1 Bowles Center for Alcohol Studies, The University of North Carolina at Chapel Hill, Chapel Hill, NC 27599, USA; 2 Carolina Institute for Developmental Disabilities, The University of North Carolina at Chapel Hill, Chapel Hill, NC 27599, USA; 3 UNC Neuroscience Center, The University of North Carolina at Chapel Hill, Chapel Hill, NC 27599, USA; 4 Department of Pharmacology, The University of North Carolina at Chapel Hill, Chapel Hill, NC 27599, USA; 5 Department of Genetics, The University of North Carolina at Chapel Hill, Chapel Hill, NC 27599, USA; 6 Department of Cell Biology and Physiology, The University of North Carolina at Chapel Hill, Chapel Hill, NC 27599, USA; 7 Carolina Institute for Developmental Disabilities, The University of North Carolina at Chapel Hill, Chapel Hill, NC 27599, USA

**Keywords:** Fetal alcohol spectrum disorders, Apoptosis, Inflammation, Embryo, Brain development

## Abstract

Genetics are a known contributor to differences in alcohol sensitivity in humans with fetal alcohol spectrum disorders (FASDs) and in animal models. Our study profiled gene expression in gastrulation-stage embryos from two commonly used, genetically similar mouse substrains, C57BL/6J (6J) and C57BL/6NHsd (6N), that differ in alcohol sensitivity. First, we established normal gene expression patterns at three finely resolved time points during gastrulation and developed a web-based interactive tool. Baseline transcriptional differences across strains were associated with immune signaling. Second, we examined the gene networks impacted by alcohol in each strain. Alcohol caused a more pronounced transcriptional effect in the 6J versus 6N mice, matching the increased susceptibility of the 6J mice. The 6J strain exhibited dysregulation of pathways related to cell death, proliferation, morphogenic signaling and craniofacial defects, while the 6N strain showed enrichment of hypoxia and cellular metabolism pathways. These datasets provide insight into the changing transcriptional landscape across mouse gastrulation, establish a valuable resource that enables the discovery of candidate genes that may modify alcohol susceptibility that can be validated in humans, and identify novel pathogenic mechanisms of alcohol.

This article has an associated First Person interview with the first author of the paper.

## Abstract (precis)

**Editor's choice:** RNA-sequencing in gastrulation-stage mouse embryos provides information about gene expression patterns during normal mouse development and evidence that pre-existing genetic variability mediates risk to prenatal alcohol-induced birth defects.

## INTRODUCTION

Alcohol exposure during the first weeks of pregnancy is associated with significant birth defects involving the craniofacial region and central nervous system (Cook et al., 1987). Specifically, prenatal alcohol exposure (PAE) during gastrulation [third week of human pregnancy; embryonic day (E)7 in mice] results in the craniofacial malformations characteristic of fetal alcohol syndrome (FAS), including a thin upper lip, smooth philtrum, reduced head circumference and small eyes (Cook et al., 1987). In addition, gastrulation-stage PAE is associated with loss of midline brain tissue, including agenesis of the corpus callosum and holoprosencephaly (Higashiyama et al., 2007; Godin et al., 2010), disrupted morphogenic signaling (Zhang et al., 2014; Kietzman et al., 2014; Aoto et al., 2008) and widespread apoptosis (Dunty et al., 2001).

An ongoing question in the field of prenatal alcohol research is why some children exposed to alcohol *in utero* develop significant physical and cognitive deficits whereas others are relatively unaffected. While the dose and timing of alcohol exposure are certainly factors, it is known that environmental factors, such as stress or nutrition, and genetics can predispose an embryo to alcohol sensitivity or resistance. Studies using twins exposed to heavy prenatal alcohol revealed that dizygotic twins were less likely to both be diagnosed with FAS compared to monozygotic twins (Streissguth and Dehaene, 1993; Abel, 1988). Of the monozygotic twins examined, if one twin was diagnosed with FAS then the other was also diagnosed in 100% of cases, compared with only 64% concordance in the dizygotic twin sets. In addition, experiments in animal models of fetal alcohol spectrum disorder (FASD) have demonstrated that strains of mice and chicken exhibit different degrees of incidence and severity of PAE-related birth defects (Downing et al., 2009; Su et al., 2001). These data clearly suggest that there is a genetic component to FAS. Although the genetic differences that alter susceptibility to PAE between these strains are not yet clear, it is known that the deletion of certain genes can alter susceptibility to PAE (Eberhart and Parnell, 2016). For example, deleting one copy of either sonic hedgehog (*Shh*) (Kietzman et al., 2014), the Shh co-receptor cell adhesion associated oncogene associated (*Cdon*) (Hong and Krauss, 2012, 2013) or downstream transcriptional activator Gli family zinc finger 2 (*Gli2*) (Fish et al., 2017) increases susceptibility to PAE in the brain, face and limbs. Likewise, deletion of one or both copies of the ciliary-related gene *Mns1* exacerbates the effects of PAE on the brain and face in a gene dose-dependent manner (Boschen et al., 2018). However, the identification of further genes that may alter susceptibility to PAE remains elusive.

In order to identify candidate genes that alter susceptibility to early developmental alcohol exposure, our current study identifies PAE-induced transcriptomic changes in the gastrulation-stage embryo using two closely related mouse strains: the C57BL/6J (referred to as 6J) strain obtained from The Jackson Laboratory and the C57BL/6NHsd (referred to as 6N) strain obtained from Envigo (formerly Harlan). Previous work has demonstrated that the 6J strain has a higher incidence of eye defects after prenatal alcohol compared to the 6N strain (Dou et al., 2013; Green et al., 2007). These strains were both derived from the original C57BL/6J mice bred by The Jackson Laboratory but were separated when the 6J strain was given to the National Institutes of Health (NIH) in 1951 and given from the NIH to Harlan in 1974. Now, over 200 generations separate the 6J and 6N strains. Notably, two known genetic mutations have emerged over the years. First, the 6J strain has a mutation in the *Nnt* gene, which encodes nicotinamide nucleotide transhydrogenase, an enzyme important for production of NADPH and removal of reactive oxygen species (ROS) from the mitochondria (Ronchi et al., 2013). The mutation in the 6J mice is comprised of two separate mutations: a missense (M35T) mutation in the mitochondrial leader sequence and a multi-exonic deletion of exons 7-11, resulting in a non-functional protein. 6J mice have been shown to have five- to sevenfold lower levels of Nnt in the islets and liver (Toye et al., 2005), impaired insulin secretion and mitochondrial redox abnormalities (Ronchi et al., 2013). Mutations in the *Nnt* gene could cause reduced NADPH and glutathione stores and impaired oxidative stress responses in the 6J embryos, possibly priming these embryos to be more likely to undergo cell death following alcohol exposure. Second, the 6N strain carries a single nucleotide deletion in the *Crb1* gene, called the *Rd8* mutation (Mattapallil et al., 2012). This mutation is associated with retinal degeneration, lesions and folding.

While the *Nnt* and *Rd8* mutation are two well-studied differences between the 6J and 6N strains, it is possible that other genetic variation is present during development that could modulate strain differences in risk and resilience to alcohol damage. In addition, it is unknown what effect these mutations have on gene expression during early embryonic development. The goals of this experiment were two pronged. First, we used the gathered transcriptome data to provide information about gene expression across gastrulation during normal mouse development. To this end, a web-based tool was created to allow gene-by-gene exploration of expression patterns across the first 12 h of gastrulation in both the 6J and 6N strains. Second, we examined PAE-induced gene expression changes 6 h and 12 h after exposure (E7.25 and E7.5, respectively), adding valuable information about the molecular targets of this mouse model of FASD.

## RESULTS

### Web-based tool as a resource for data visualization and exploration

**Fig. 1.**
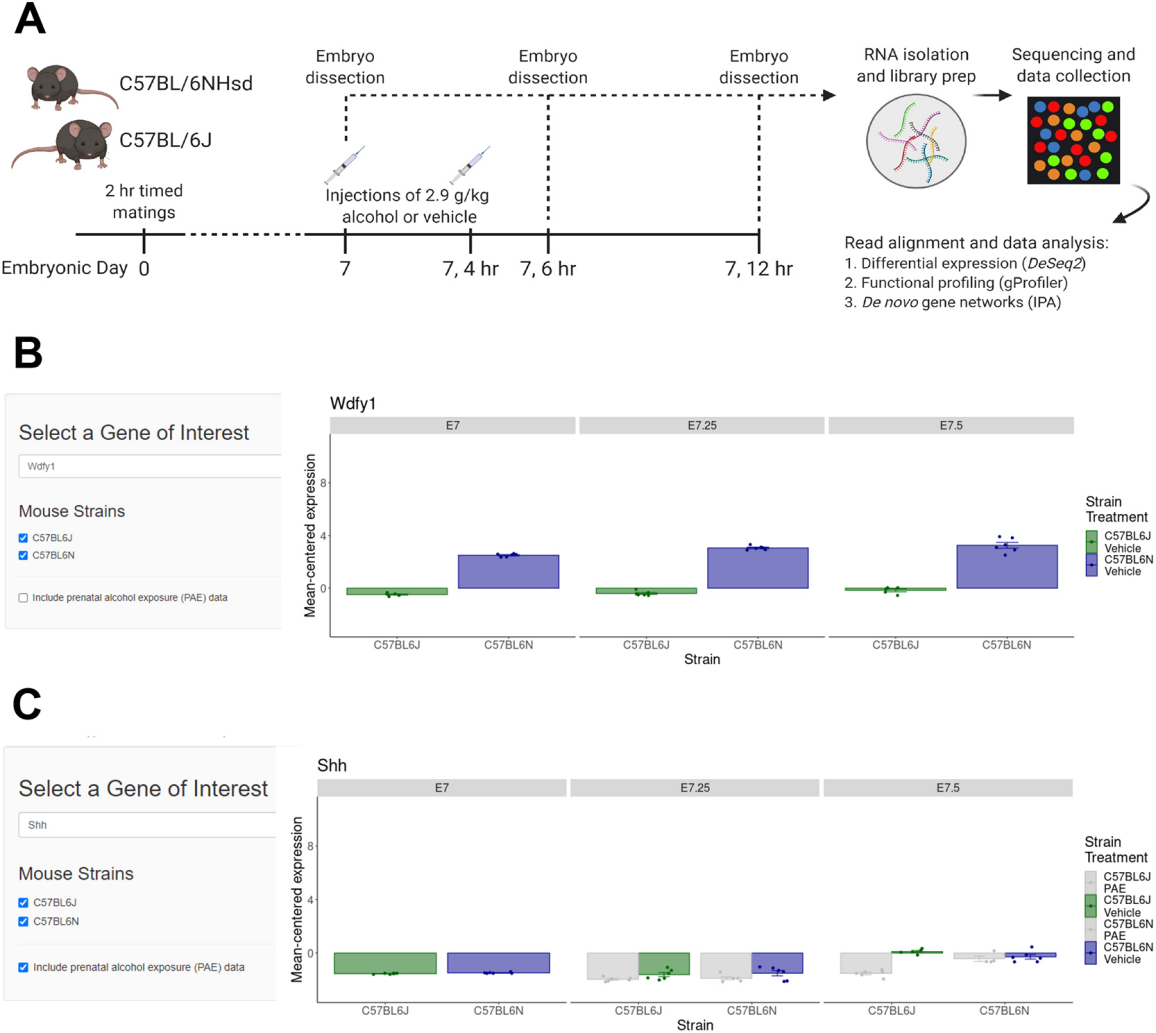
**Experimental timeline and example of web-based visualization tool.** A web tool was created as a resource to allow gene-by-gene exploration of expression patterns across the first 12 h of normal mouse gastrulation in the 6J and 6N strains. (A) Experimental timeline. (B) Comparison of expression of *Wdfy1*, a gene that significantly differed between the 6J and 6N strains, across time. Single strains can be selected for viewing using the toggles on the left. (C) PAE data can be toggled on and off using the options. *Shh* expression was impacted by prenatal alcohol exposure (PAE) in the 6J, but not 6N, mice. Figure created with Biorender.com.

We performed whole transcriptomic analyses of 6J and 6N mouse embryos at three time points (E7.0, E7.25 and E7.5) using RNA sequencing (RNA-seq) (Fig. 1A). We assembled a transcriptomic database of normal embryonic development, as well as characterized how strain differences and PAE treatment governs these processes. A web-based visualization tool (http://parnell-lab.med.unc.edu/Embryo-Transcriptomics/) was created for a gene-by-gene query of the transcriptomic data from both strains and from both control and PAE-treated embryos at each time point. Strain and prenatal treatment options can be toggled on or off to compare relative expression of a gene of interest in a single strain across time points, or between the 6J and 6N strains across time. For example, expression of *Wdfy1* significantly differs between the strains across all time points but is not affected by PAE (Fig. 1B). Conversely, *Shh* increases in expression in both strains over time, but PAE significantly reduces expression in the 6J strain (Fig. 1C). The gene expression data generated in this study provide a valuable resource for developmental biologists, toxicologists, mouse geneticists and researchers interested in models of FASD.

### Transcriptional differences between 6J and 6N mouse embryos during gastrulation

**Fig. 2.**
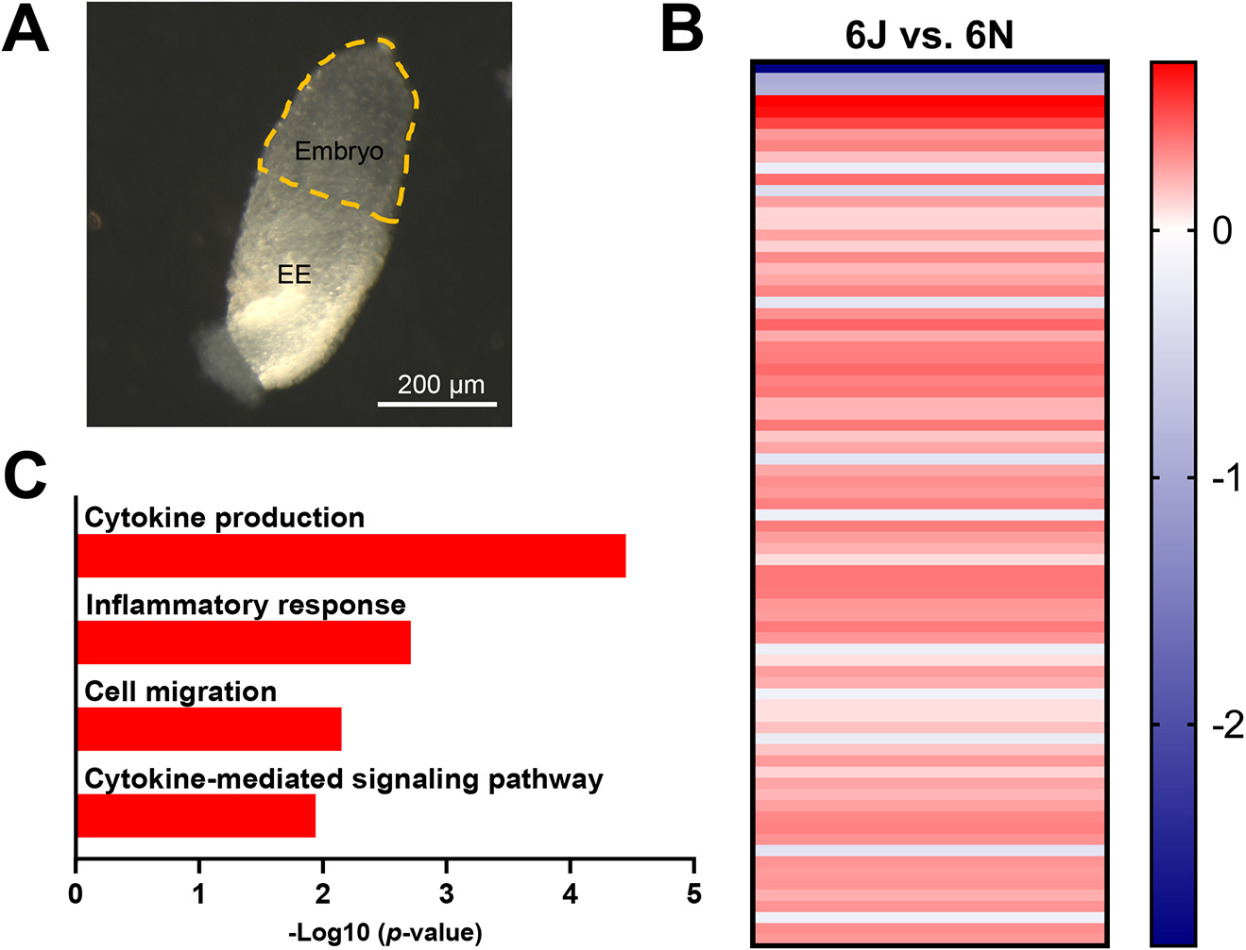
**Immune signaling gene pathways are upregulated in the 6J compared to the 6N strain.** (A) Representative image of E7.0 mouse embryo. Embryo highlighted in yellow was dissected from the extraembryonic tissue (EE) for sequencing. (B) Heat map of genes altered in the 6J versus 6N strain at baseline (prior to alcohol administration) on E7.0. Data are expressed as log_2_ fold change (Log_2_FC). Blue, downregulated genes; red, upregulated genes. *n*=6/group. (C) Functional profiling of genes differentially expressed in the 6J versus 6N strain at E7.0. *n*=6/group.

Gene expression across the first 12 h of normal mouse gastrulation was compared between the 6J and 6N strains (a representative image of a gastrulation-stage mouse embryo is shown in Fig. 2A). Heat maps showing hierarchical clustering of gene expression of all significant genes for all replicates are in Figs S1-S7, and VST-normalized values for all significant genes are in Dataset 1. We first focused on how 6J and 6N embryonic gene expression differs at E7.0 to establish a baseline and explore strain-dependent transcriptional differences prior to alcohol exposure. Eighty genes were identified as differentially expressed between the 6J and 6N strains at E7.0. Of these, 67 showed higher expression (83.8%) and 13 showed lower expression (16.2%) in the 6J relative to 6N strain (Fig. 2B). Functional profiling revealed upregulation of pathways related to inflammation and cytokine production, cell migration and intracellular signaling (Fig. 2C; Table S1).

Multiple genes that encode cytokines/chemokines and immune signaling molecules had higher expression in the 6J strain, including *Ccl4*, *Il1r1*, *Il1rn* and *Tnfrsf9*. The most upregulated gene [largest positive log_2_ fold-change (Log_2_FC)], *Ide*, encodes an insulin-degrading enzyme that is known to degrade the B chain of insulin and amyloid beta (Bennett et al., 2000), suggesting a role in Alzheimer's disease. Expression of *Ide* has been found to be relatively low in embryonic *Drosophila* (Stoppelli et al., 1988) and neonatal rat (Kuo et al., 1993) compared to their adult counterparts, suggesting a more prevalent role of this protein during adulthood. There were no significantly overenriched pathways among the downregulated genes; however, the *Nnt* gene was significantly downregulated in the 6J strain, corroborating the well-known mutation in the 6J mouse strain (Ronchi et al., 2013). The gene most downregulated in the 6J relative to 6N strain was *Wdfy1*, which encodes an adaptor protein involved in protein–protein and protein–DNA interactions. Wdfy1 also acts as an adaptor protein for Toll-like receptors 3 and 4 (Hu et al., 2015), implicating this protein in the immune signaling response. *Efcab7* was also downregulated in the 6J relative to 6N strain; this gene is associated with primary cilia function and, in particular, Shh signaling via smoothened (Smo) (Pusapati et al., 2014).

Because the majority of pathways and genes were related to the cellular immune signaling response, we hypothesized that 6J mice have heightened immune signaling activity that influences the stress response to a stimulus such as alcohol. We therefore sought to more comprehensively characterize the disrupted *de novo* gene networks using the Ingenuity Pathway Analysis (IPA) database of known protein–protein interactions. IPA allows insight into the functional relationships between differentially expressed genes that are not captured in the canonical terms and pathways used in the gene set enrichment analysis above. Six networks were dysregulated in the 6J relative to 6N (Table 1A; Table S2A) related to immune signaling (‘Inflammatory disease’, ‘Immune cell trafficking’, ‘Inflammatory response’) and cell proliferation (‘Cell cycle’, ‘Cell movement’, ‘Cellular assembly and organization’), supporting that baseline immune signaling differs between the strains. Differences in cell movement are likely to be linked to immune cell migration, although the source, type and function of these immune cells and related signaling molecules in the gastrulation-stage embryo is not yet clear. Overall, these genetic differences set the stage for the disparate responses to PAE observed in these two strains both hours (Figs 4 and 6) and days (Dou et al., 2013; Green et al., 2007) later in development.

**Table 1.**
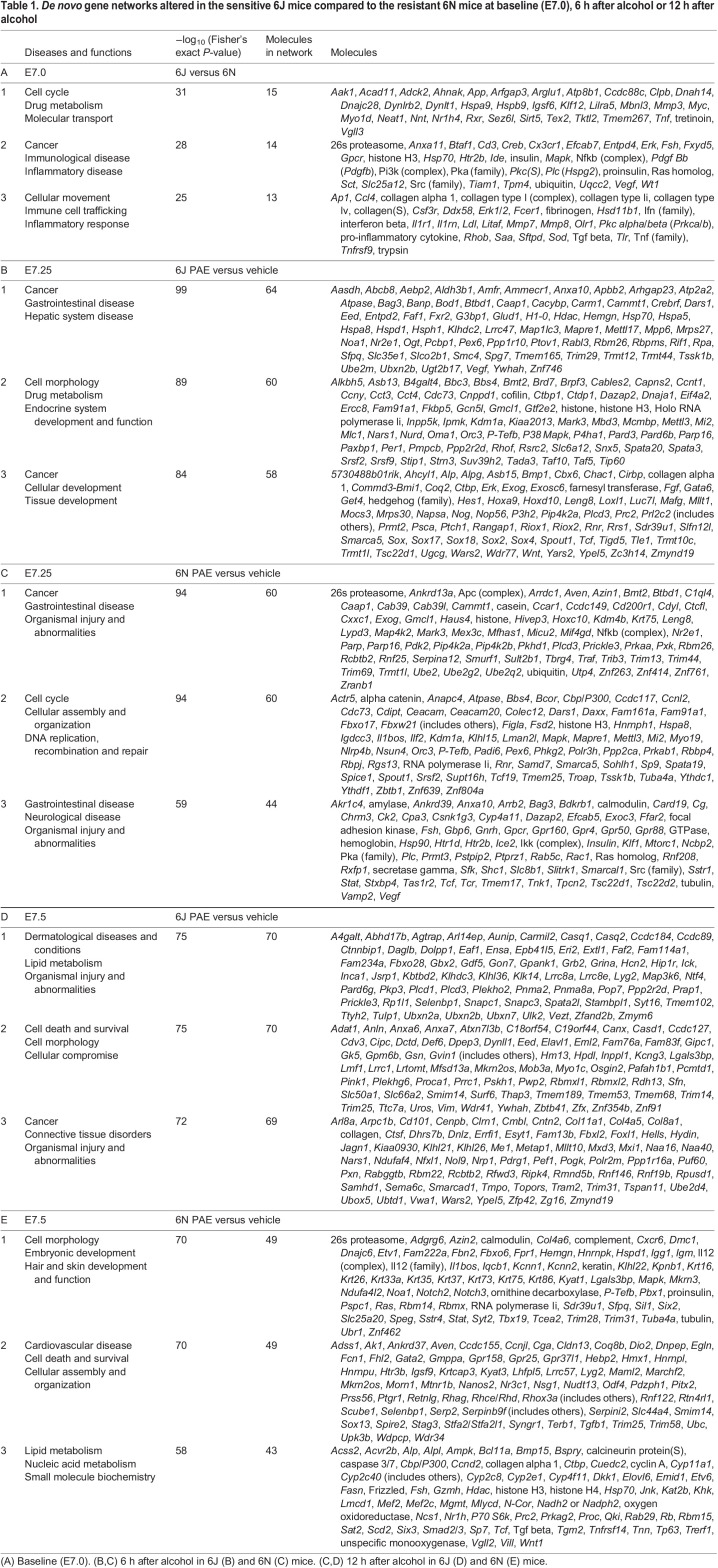
*De novo* gene networks altered in the sensitive 6J mice compared to the resistant 6N mice at baseline (E7.0), 6 h after alcohol or 12 h after alcohol

We next compared the 6J and 6N strains either 6 h or 12 h after E7.0 following two injections of vehicle solution to investigate strain differences at later developmental time points in the absence of alcohol. Gastrulation is a critical time of embryonic development, involving cell proliferation and fate decisions that establish the embryonic germ layers, with developmental events relying on temporally and spatially specific gene expression (Pijuan-Sala et al., 2019). At E7.25, 6 h post-vehicle injection, 315 genes were differentially expressed between the two strains. Of these genes, 128 genes were upregulated (40.6%) and 187 were downregulated (59.4%) in the 6J relative to the 6N strain. Twelve hours after vehicle treatment, at E7.5, there were 304 differentially expressed genes between the 6J and 6N strains. Of these, similar to the E7.25 time point, 120 genes were upregulated (39.5%) and 184 genes were downregulated (60.5%) in the 6J strain.

Functional profiling of genes from the E7.25 time point revealed only a small number of biological pathways that differed between the two strains, including altered hydrolase and endopeptidase activity and pathways related to cAMP signaling and apoptosis related to the downregulated genes (Table S3). The top ten *de novo* networks were related to cell death, intercellular signaling, nutrient metabolism and embryonic development (Table S2B). At E7.5, functional profiling of the upregulated genes indicated increased prostaglandin signaling and GPCR signaling (Table S4). The downregulated pathways were again related to hydrolase and endopeptidase activity, consistent with the E7.25*. De novo* network analysis identified functions related to organ development, drug metabolism, protein processing and the cell cycle (Table S2C).

Overall, there was little change in which genes were strongly up- or downregulated across these 12 h of development, consistent with other studies showing that most genes expressed during gastrulation show relatively stable expression prior to the onset of organogenesis (Mitiku and Baker, 2007). *Wdfy1* showed the largest downregulation by Log_2_FC at both time points in the 6J relative to 6N strain (−3.94 and −3.67 Log_2_FC, respectively), consistent with what was observed in these two strains at E7.0 prior to any injection. *Efcab7*, which had lower baseline expression in the 6J strain, exhibited the same effect at E7.25 (−1.78 Log_2_FC), but not at E7.5. The most upregulated gene in the 6J relative to 6N strain at E7.25 was *Hist1h4m* (*H4c17*), or histone cluster 1, H4m, a gene related to nucleosome assembly. This gene also showed a large upregulation at E7.5 and a small but statistically significant upregulation at E7.0. Interestingly, this gene was found to be downregulated in the hippocampus of fetal 6J mice (purchased from Orient Bio) following alcohol exposure from E8 to E12 (Mandal et al., 2015). The fact that expression of *Hist1h4m* differs between alcohol-sensitive and -resistant strains and expression is affected in certain models of PAE is strongly suggestive that this gene is a possible target of alcohol and mediator of alcohol sensitivity.

### Strain-specific differences in transcriptional response to PAE are evident as early as 6 h after exposure

**Fig. 3.**
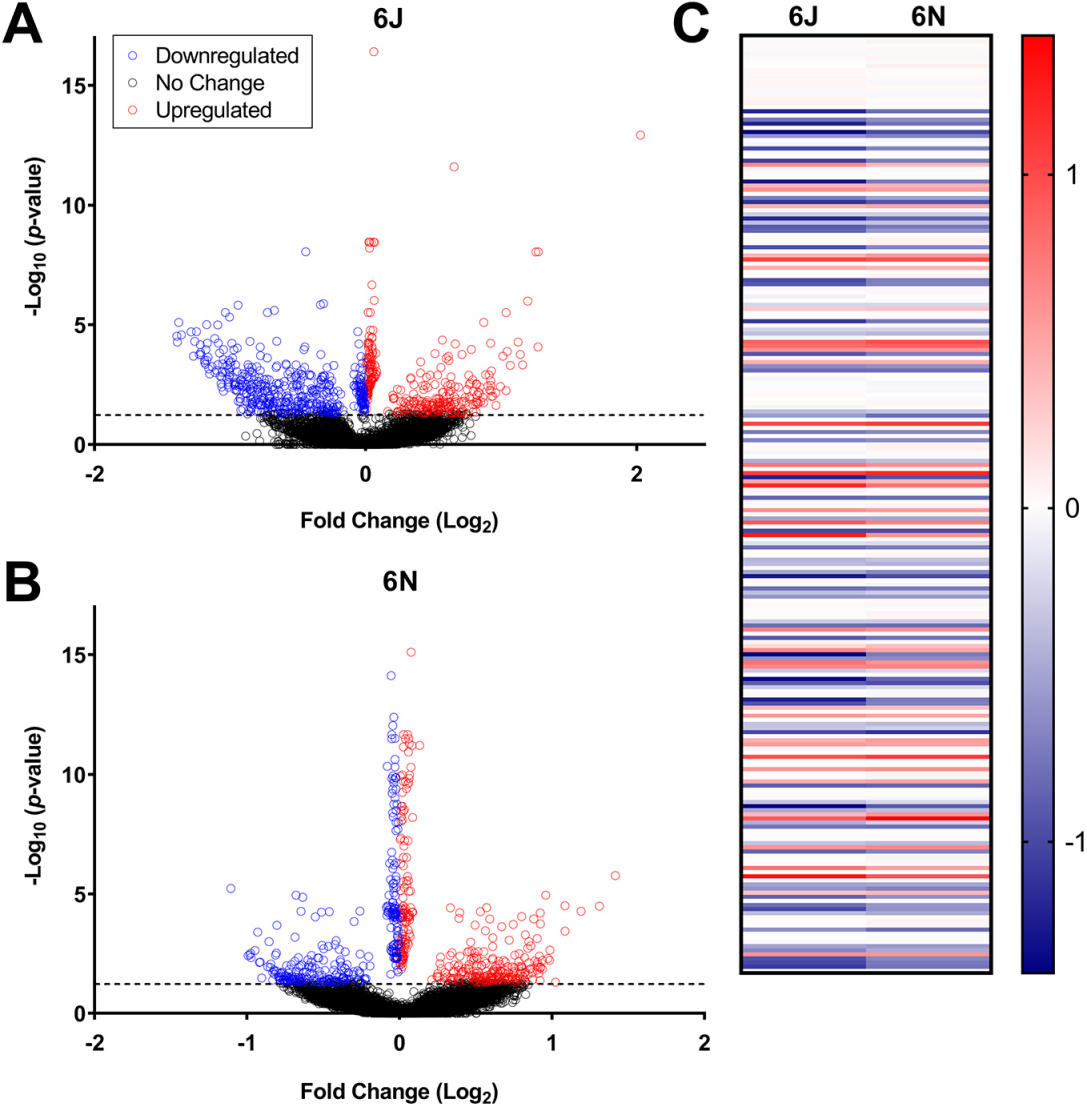
**Gastrulation-stage alcohol dysregulated more genes in the 6J strain than** **in** **the 6N strain 6 h after exposure.** (A) Volcano plot of genes significantly dysregulated by alcohol in the 6J mice 6 h after the first dose of alcohol (E7.25). (B) Genes significantly dysregulated by alcohol in the 6N mice 6 h after the first dose of alcohol (E7.25). (C) Heat map comparing 228 genes altered by alcohol in both the 6J and 6N mice at the E7.25 time point. Data are expressed as Log_2_FC. Blue, downregulated genes; red, upregulated genes. *n*=6/group.

We next compared the effect of PAE on embryonic gene expression in each strain at E7.25 to explore how strain differences modulate the initial transcriptional response to alcohol. At E7.25 (6 h post-PAE), 810 genes were significantly differentially expressed between PAE and vehicle in the 6J strain, and 702 genes were differentially expressed between PAE and vehicle in the 6N strain. In the 6J strain, 355 genes were upregulated (43.8%) and 455 were downregulated (56.2%) (Fig. 3A). In the 6N strain, 372 genes were upregulated (52.9%) and 330 were downregulated (47.1%) (Fig. 3B). Of the differentially expressed genes, 228 were altered in both strains (Fig. 3C). In most cases, the directionality (up- or downregulated) was the same between strains, indicating that although there is a substantial subset of genes that are similarly affected in both the 6J and 6N strains, the majority of genes with significantly altered expression in each strain are unique.

**Fig. 4.**
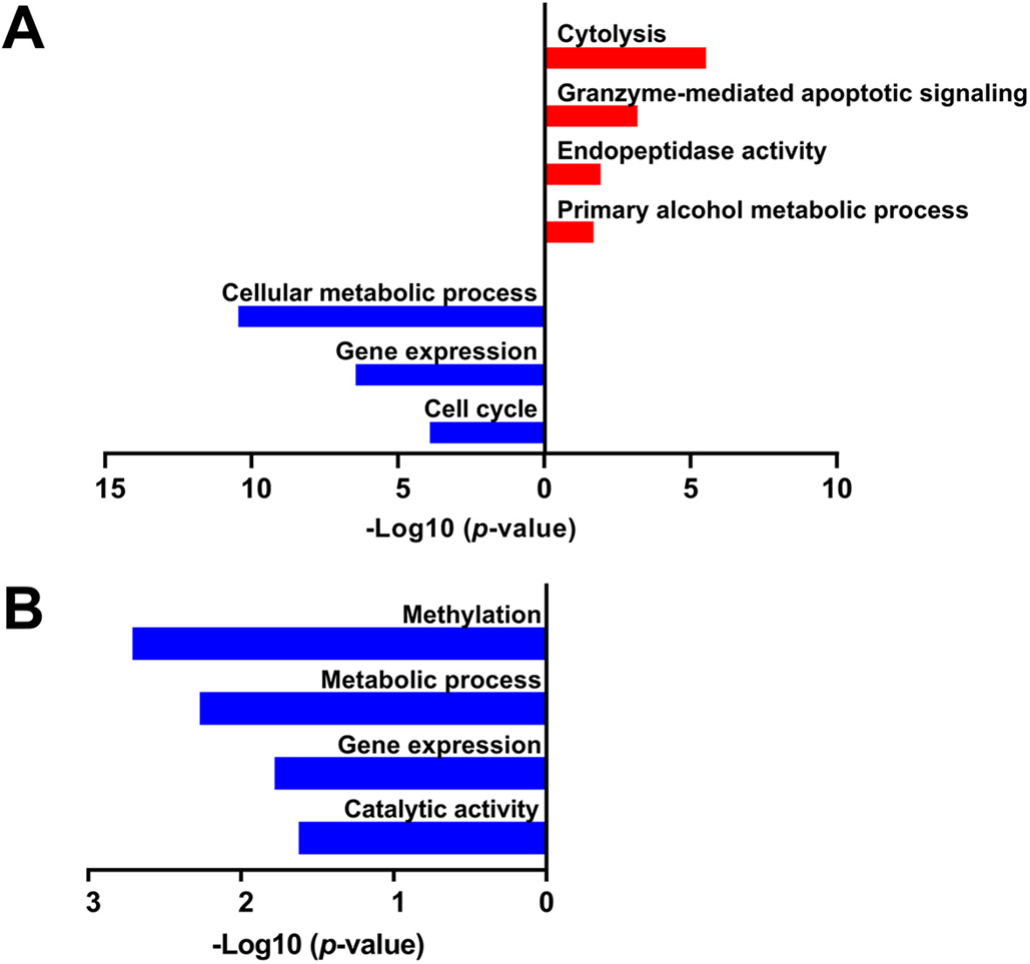
**Functional profiling of biological pathways enriched in the 6J and 6N strains 6 h after alcohol exposure (E7.25).** (A) 6J strain. (B) 6N strain. *n*=6/group.

Functional profiling of the genes upregulated following PAE in the 6J strain at E7.25 revealed that pathways related to catalytic activity (specifically, hydrolases and endopeptidases) were dysregulated (Fig. 4A; Table S5). Interestingly, we identified ‘Activation, myristoylation of BID and translocation to mitochondria’ as upregulated by PAE. BH3-interacting domain death agonist (BID) is a pro-apoptotic protein of the Bcl-2 family that is activated by the post-translational modification N-myristoylation. Activation of BID causes the insertion of Bax into the mitochondrial membrane and release of cytochrome C (Eskes et al., 2000). This pathway, in combination with others related to cytolysis and apoptotic signaling, indicates that cell death pathways have begun to be activated in the 6J strain as early as 6 h post-PAE (E7.25). Analysis of downregulated genes in the 6J strain found that cellular metabolism and binding activity were reduced. ‘Binding activity’ included enzymatic, DNA and protein binding, and likely indicates an overall reduction in cellular activity that coincides with decreased metabolism. Multiple terms related to cell cycle regulation were also identified in the downregulated genes, suggesting that cell proliferation is slowed or paused while the embryo responds to the alcohol insult. *De novo* network analysis revealed multiple associations with organ health and development, cancer/cell cycle, drug metabolism and cell death (Table 1B; Table S2D).

The top two genes downregulated following PAE in the 6J strain at E7.25 were *Srsf2*, which encodes a protein that regulates constitutive and alternative splicing of pre-mRNA that has been linked to cell death through the p53 pathway (Comiskey et al., 2020), and *Alyref*, which encodes the molecular chaperone Aly/REF export factor, which is involved in RNA processing and nuclear export. The most upregulated gene in the 6J strain was *Chac1*, which encodes glutathione-specific gamma-glutamylcyclotransferase-1 (Gamma-GCG1), a protein involved in glutathione cleavage, induction of oxidative stress-related apoptosis, and a negative regulator of Notch signaling (Chi et al., 2012). *Trib3* was also significantly upregulated and encodes Tribbles pseudokinase 3 (Trb-3), which is induced by NF-κB signaling, creates a negative feedback loop controlling Atf4 activity in response to cellular stress and prevents apoptosis. Interestingly, Trb-3 has also been shown to block expression of Gamma-GCG (Örd et al., 2016), thus limiting apoptosis through another pathway. Multiple types of cellular stress upregulated Trb-3, including nutritional deprivation (Liu et al., 2012) and endoplasmic reticulum (ER) stress (Örd et al., 2014). In sum, alcohol-induced reductions in the expression of cellular metabolism and gene transcription pathways, as well as upregulation of genes related to oxidative stress and apoptosis, could lead to perturbed cell proliferation and embryonic growth in PAE 6J embryos.

We next compared gene expression patterns following PAE in 6N mice. Although 372 genes were upregulated, there were no significantly enriched pathways among them. However, analysis of the 330 downregulated genes revealed a reduction in cellular metabolism and methyltransferase activity (Fig. 4B; Table S6). Similarly to in the 6J strain, PAE seemingly caused a reduction in cellular activity in the 6N strain. Alteration of methylation could have effects on gene expression and protein function; some of the specific methyltransferases targeted by PAE included *Kdm1a*, *Kdm4b*, *Mettl3*, *Mettl4*, *Mettl16* and *Prdm5*, among others. Gene network analyses also revealed pathways related to organ and tissue disease, cell cycle/DNA replication and repair, and cell and tissue morphology (Table 1C; Table S2E). The two most downregulated genes were *Rsrp1*, which encodes the relatively unknown protein arginine/serine rich protein 1, a target of heat shock protein 1 under certain conditions (Korfanty et al., 2014), and *Alyref*, described above. *Tap2*, a transporter protein involved in multi-drug resistance and antigen presentation through localization of peptides to the ER, where they are then transported to the cell surface, and *Sox15*, a member of the Sox family, were the two most upregulated genes. The Sox family is comprised of transcription factors that play vital roles in embryonic development and specification of cell fate. Sox15 expression is highest in undifferentiated embryonic stem cells (Maruyama et al., 2005), suggesting that PAE may disrupt cell differentiation in 6N mice, resulting in increased expression of *Sox15*. Overall, although PAE causes a reduction in cellular activity that could disrupt proliferation and cell fate decisions, there is no evidence that cell death pathways are activated at this point in the 6N strain, a notable difference from the 6J strain.

### Large-scale strain-specific differences in transcriptional response to PAE apparent 12 h after exposure

**Fig. 5.**
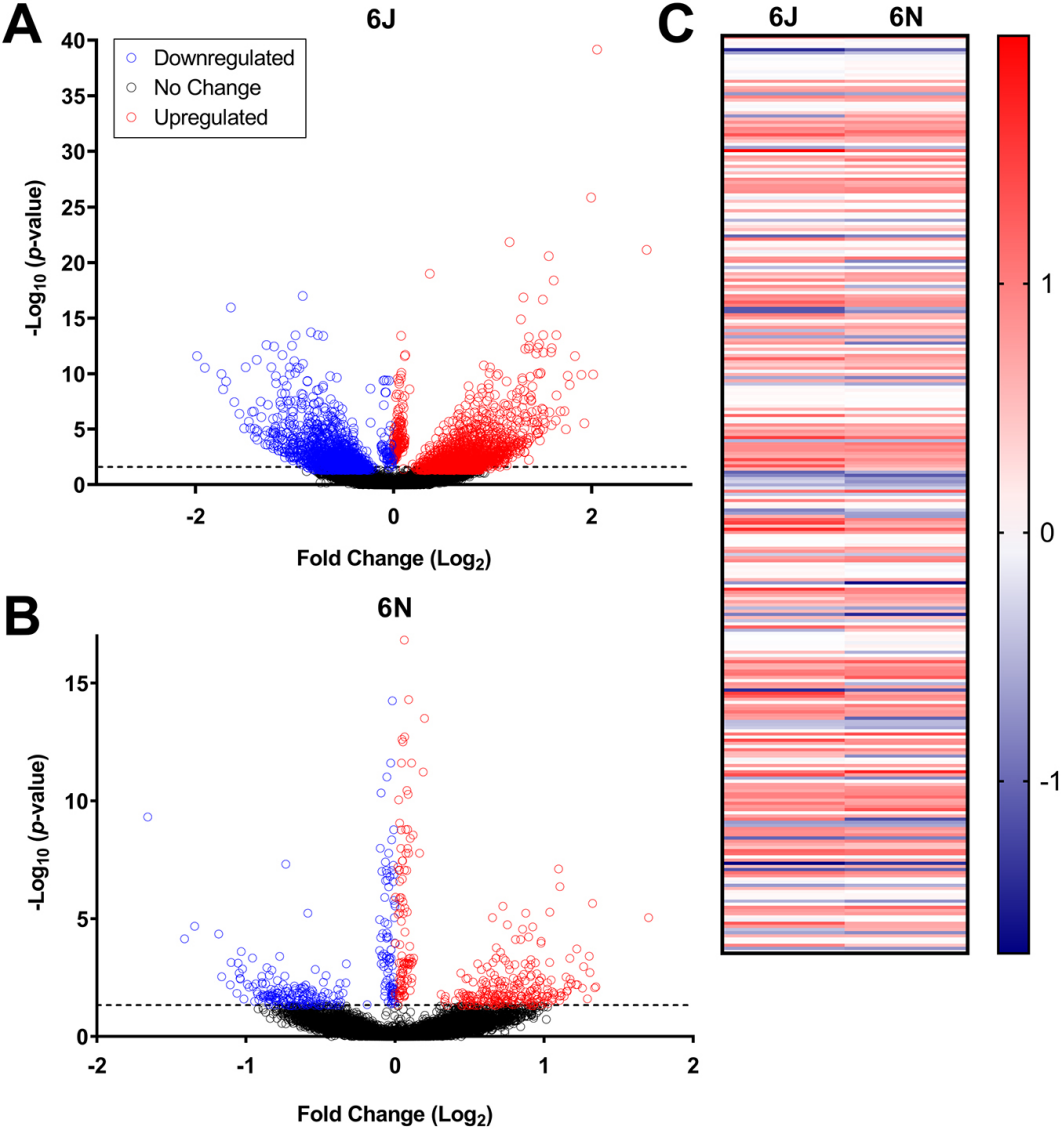
**Gastrulation-stage alcohol dysregulated more genes in the 6J strain than** **in** **the 6N strain 12 h after exposure.** (A) Volcano plot of genes significantly dysregulated by alcohol in the 6J mice 12 h after the first dose of alcohol (E7.5). *n*=5 vehicle-treated, *n*=6 PAE. (B) Genes significantly dysregulated by alcohol in the 6N mice 12 h after the first dose of alcohol (E7.5). *n*=6 vehicle-treated, *n*=4 PAE. (C) Heat map comparing 228 genes altered by alcohol in both the 6J and 6N mice at the E7.5 time point. Data are expressed as Log_2_FC. Blue, downregulated genes; red, upregulated genes. *n*=6/group.

To explore how strain differences continue to modulate the transcriptional landscape 12 h after alcohol exposure, we next compared the effect of PAE on embryonic gene expression in each strain at E7.5. At E7.5 (12 h post-PAE), the 6J strain continued to have more pronounced gene expression changes relative to the 6N strain; in fact, the number of differentially expressed genes increased over threefold in the 6J strain, whereas it remained relatively stable in the 6N strain. In the 6J strain, 2987 genes were differentially expressed 12 h after PAE. Of these, 1641 were upregulated (54.9%) and 1346 were downregulated (45.1%) (Fig. 5A). Conversely, only 641 genes were altered by PAE in the 6N strain at this time point, with 366 upregulated (57.1%) and 275 downregulated (42.9%) (Fig. 5B). The significant increase in the number of differentially expressed genes in the 6J but not the 6N strain provides further evidence that 6J mice are more sensitive than 6N mice to PAE; 291 genes in total overlapped between the two strains (Fig. 5C). While most genes significantly altered by PAE in both strains showed the same direction of change, most of the overlapping genes were upregulated at E7.5, compared to most overlapping genes being downregulated in both strains at E7.25.

**Fig. 6.**
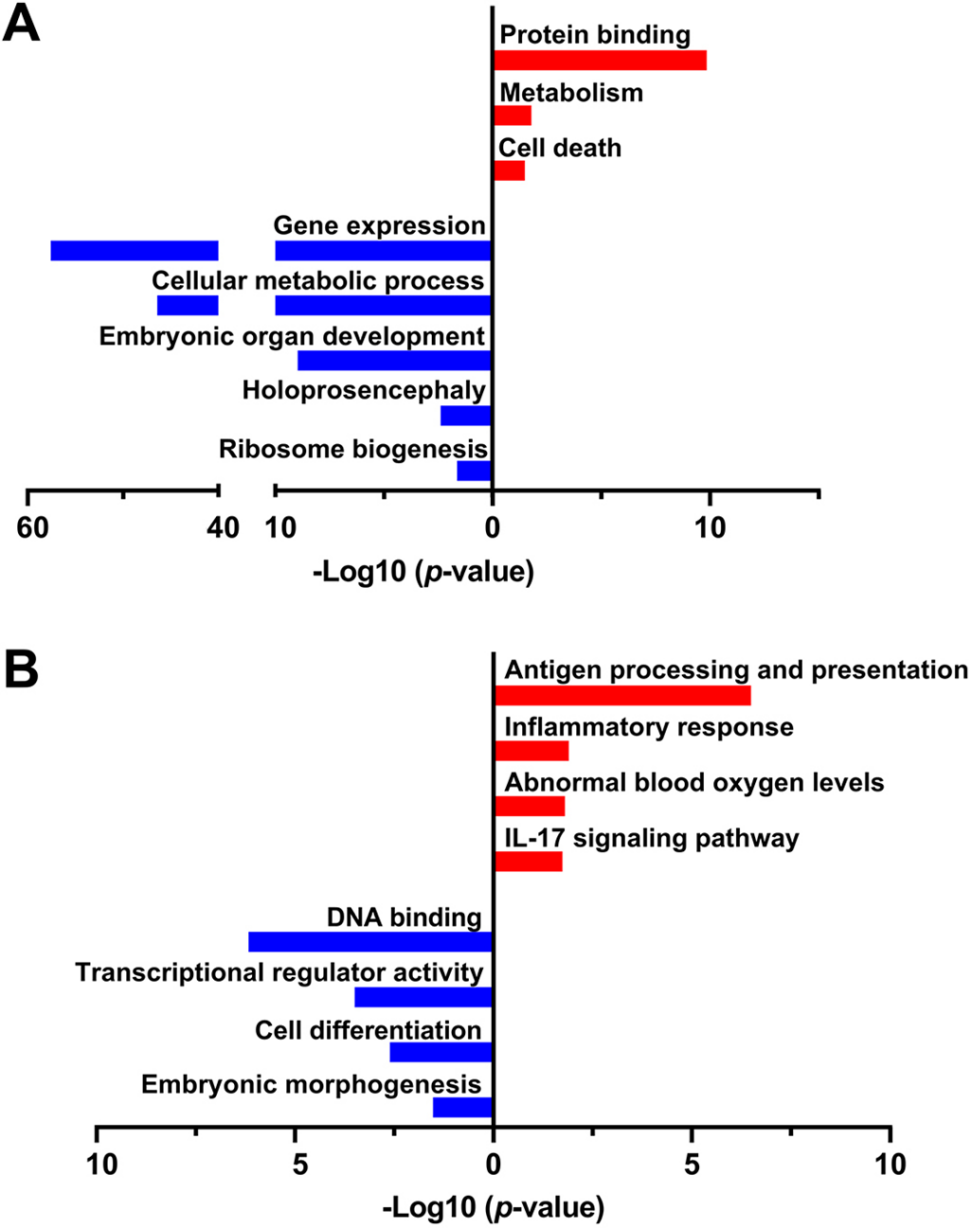
**Functional profiling of biological pathways enriched in the 6J and 6N strains 12 h after alcohol exposure (E7.5).** (A) 6J strain. (B) 6N strain. *n*=6/group.

Functional profiling of the genes upregulated 12 h following PAE in 6J mice revealed pathways related to intracellular signaling, protein transport and localization, and cell death (Fig. 6A; Table S7). One of the upregulated pathways we identified in 6J mice was ‘Formation of xylulose-5-phosphate’. Xylulose-5-phosphate is a ketose sugar known to promote gene t­ranscription through the ChREBP transcription factor (encoded by *Mlxipl*). This was interesting because *Mlxipl* was itself significantly upregulated in the 6J mice at this time point. ChREBP is part of the Myc superfamily and has been found to affect cell proliferation through regulation of transcription of cyclins in certain cell types (Filhoulaud et al., 2013; Tong et al., 2009), although its exact function in early gestational embryos is not known. In addition, ChREBP has multiple isoforms and can be stored in an inactive form. The downregulated genes in the 6J mice were enriched for embryonic organogenesis and skeletal development, including the head, palate and circulatory system. Notably, holoprosencephaly, cleft palate, and abnormal lip, ear and face shape were identified using the Human Phenotype Ontology (HPO) database as phenotypes associated with PAE. These craniofacial malformations have been associated with heavy alcohol exposure during early gestation in the human population (DeRoo et al., 2008; Romitti et al., 2007; Johnson and Rasmussen, 2010; Jones et al., 2010). Analysis of *de novo* gene networks found differences in pathways related to organismal injury and abnormalities, cell death, organ disease, embryonic development, and protein and RNA post-translational modifications of RNA and proteins (Table 1D; Table S2F).

The most downregulated gene in the 6J strain at E7.5 was *Shh*. Multiple other members of the Shh pathway were also downregulated by PAE in the 6J strain at this time point, including *Ptch1*, *Smo* and *Gli3*. In contrast, *Gli3* was the only member of the pathway affected by alcohol in the 6N mice at either time point. Dysregulation of the Shh pathway is linked to craniofacial malformations such as holoprosencephaly in genetic ciliopathies (Brugmann et al., 2015; Chang et al., 2016), and PAE both downregulates Shh expression (Higashiyama et al., 2007) and causes more severe craniofacial and limb defects in transgenic mice lacking genes in the Shh pathway (Fish et al., 2017; Kietzman et al., 2014). If alcohol is more likely to impact Shh signaling in the 6J than the 6N strain, this presents one way in which 6J may be more likely to develop craniofacial and eye defects. Whether there is an association between the higher baseline expression of immune genes in the 6J strain and differences in Shh signaling after alcohol exposure is not yet clear, but these findings warrant further exploration. *Efcab7*, a gene linked to Smo transduction in the primary cilia (Pusapati et al., 2014), was upregulated by PAE at E7.5 (+0.81 Log_2_FC). This gene had lower expression in the 6J strain relative to the 6N strain at E7.0 and E7.25, suggesting possible pre-existing differences between the strains; however, more work needs to be done to determine the exact role of Efcab7 during gastrulation and, in particular, in relation to Shh signaling. Another downregulated gene, *Tcf21*, encodes transcription factor 21, a protein with varied and important functions during lung, kidney, heart and gonadal development (Braitsch et al., 2012; Quaggin et al., 1999; Tamura et al., 2001). Downregulation of the *Shh* pathway and *Tcf21* suggest serious and widespread defects in organogenesis in the 6J mice just 12 h following PAE, an effect that does not seem to occur in the 6N mice.

The top two upregulated genes in the 6J strain were *Tap2* and *Fam46b* (*Tent5b*), the first of which was also one of the top upregulated genes in the 6N strain at E7.25. Fam46b (TENT5B in humans) has recently been shown to be highly expressed in undifferentiated embryonic stem cells, with a sharp drop in expression following cell differentiation (Hu et al., 2020). While its biological functions remain to be fully elucidated, particularly in the embryo, Fam46b could play a role in cell cycle regulation as it inhibits cell proliferation in *in vitro* models of prostate cancer (Liang et al., 2018). Overall, these data indicate that PAE has a profoundly damaging effect in the 6J strain that is apparent within 12 h of exposure. In addition to the downregulation of Shh pathway genes, multiple genes regulating the p53 pathway were also dysregulated, including *Hif1a*, *Mdm2*, *Sirt1* and *Sco2*, indicating that cell proliferation, DNA damage repair mechanisms, cell cycle regulation and apoptosis are among the primary targets modulated by PAE in this strain. These data establish an association between baseline genetic variations between strains that lead to more deleterious outcomes in response to alcohol exposure.

Analysis of upregulated genes in the 6N strain indicated that PAE caused an increased inflammatory signaling response in these embryos compared to controls, as well as catalytic activity and RAGE (AGER) receptor binding (Fig. 6B; Table S8). Increased Il17 signaling was also identified as an upregulated pathway in this dataset, further supporting that PAE is causing immune signaling activation, which could have downstream effects on cell survival and tissue growth. Multiple phenotypes related to hypoxemia were found to be upregulated in the 6N strain using the HPO database, indicating that PAE could be affecting cellular oxygen levels up to 12 h later. Analysis of downregulated genes in the 6N strain revealed pathways related to overall cellular activity, DNA binding, and skeletal and neuronal development. Network analysis revealed that pathways related to cell morphology, embryonic development, cell death, cellular metabolism and inflammation were also altered by PAE in the 6N strain at E7.5 (Table 1E; Table S2G).

The top downregulated genes in the 6N strain at E7.5 were *Mef2c* and *Nkx2-5*. *Mef2c* encodes the transcription factor myocyte enhancer factor 2C (Mef2c) important for skeletal muscle and central nervous system (CNS) development. Humans with mutations in *MEF2C* exhibit severe intellectual disabilities, loss of muscle tone, mild craniofacial dysmorphologies and severe seizures. Transgenic mice with knockout of *Mef2c* display disorganized vasculature and cardiovascular defects. *Nkx2-5* encodes NK2 homeobox 5, known to be involved in heart development and highly expressed in the cardiac crescent cells at E7.5. Knockdown of this gene is embryonically lethal at ∼E9-E10 and causes growth retardation and heart defects.

The top two upregulated genes were *S100a9* and resistin-like gamma (*Retnlg*). S100a9 is a damage-associated molecular pattern molecule (DAMP) that makes a heterodimer with S100a8 to create calprotectin, a protein complex that produces pro-inflammatory activity when secreted from neutrophils, although cells from a neutrophil lineage are not known to be present in the embryo during gastrulation (McGrath et al., 2014). Increased concentrations of extracellular S100a9 and S100a8 induce apoptosis and stimulate ROS production in certain cell types (Lim et al., 2011). S100a9 is also known to interact with the RAGE receptor pathway, a part of the innate immune system and a primary receptor for Hmgb1, a protein previously shown to be part of the inflammatory response to alcohol in the adolescent and adult brain (Coleman et al., 2018; Vetreno and Crews, 2012). The function of *Retnlg* is largely unexplored, although it shares some similarity with human resistin (RETN), a hormone released by adipose tissue.

In summary, while PAE affects pathways related to embryonic development in the 6N strain, these pathways do not seem to be as clearly linked to craniofacial development as those identified in the 6J strain, possibly contributing to the phenotypic differences observed between these strains following PAE.

### Limited overlap in PAE-induced transcriptional differences between the 6J and 6N strains 6-12 h after exposure

Only seven genes were differentially expressed following PAE in both strains at both time points (Fig. S8). Three of these genes – *Aven*, *Hist3h2a* and *Tbx1* – were strongly downregulated in both strains at both time points. *Aven* encodes the cell death regulator Aven protein, which inhibits apoptosis through suppression of pro-apoptotic Apaf1 and augmentation of anti-apoptotic BCL-X_L_ activity and regulates the G2/M DNA damage checkpoint during cell cycle progression (Gross, 2008). Interestingly, this gene was also downregulated in the rostroventral neural tube of 6J mice 6 h after neurulation-stage alcohol exposure in a previous study (Boschen et al., 2020), revealing this gene as a marker of PAE across multiple models of FASD. The next gene, *Hist3h2a* (*H2aw*), is translated into a core component of chromatin, histone H2A cluster 3. Chromatin dynamics regulate access of transcription factors to the DNA and control processes such as cell proliferation and differentiation. *Hist3h2a* was also found to be downregulated by neurulation-stage alcohol in a whole-embryo culture model derived from C57BL/6J mice (Zhou et al., 2011). The third downregulated gene, *Tbx1*, encodes Tbox-1, a well-studied transcription factor important for cell proliferation during embryonic development. Loss of Tbx1 function is associated with 22q11 deletion/DiGeorge syndrome phenotypes, including heart defects, craniofacial abnormalities and cleft palates (Jerome and Papaioannou, 2001; Verdelli et al., 2017; Yagi et al., 2003). One of the genes that was upregulated in both strains at both time points was *Sdr39u1*, which encodes a short-chain dehydrogenase with oxidoreductase activity localized to the mitochondria and is thought to have a binding site on NADP. This protein has been identified as a possible biomarker candidate for neurodegenerative diseases such as Alzheimer's disease (Rahman et al., 2020), although its exact function is still under scrutiny. While there is little evidence directly linking Sdr39u1 to the oxidative stress response, production of NADP is a key player in cellular antioxidation.

## DISCUSSION

Understanding variables that modulate prenatal alcohol sensitivity has been an important area of research given the well-known variability of outcomes in children exposed to alcohol *in utero* and in animal models of FASD. The wide range of signs and symptoms of PAE present problems not only for the diagnosis and treatment of individuals with FASDs, but for a complete understanding of the pathogenic mechanisms of alcohol. The current study adds valuable information regarding the contribution of genetics to prenatal alcohol susceptibility by demonstrating that baseline genetic differences between two closely related mouse substrains can result in significantly different molecular responses to a teratogen such as alcohol. While only 80 genes differed between the alcohol-sensitive 6J strain compared to the 6N strain at E7.0, the 6J strain had significantly more genes dysregulated by alcohol 6-12 h later. Functional profiling also revealed that the biological functions affected by alcohol in the 6J mice differed from those identified in the 6N mice. Gene expression pathways related to cell proliferation, apoptosis, and those controlling craniofacial and brain development were affected in the 6J embryos. In contrast, cellular metabolism, hypoxemia, and inflammation pathways were altered in the 6N embryos. Overall, these data indicate that gastrulation-stage alcohol exposure might alter cell proliferation in both strains, but apoptosis pathways are more strongly enriched in the 6J strain, likely contributing to the increased incidence of eye defects following PAE in the 6J compared to the 6N fetuses (Dou et al., 2013).

**Fig. 7.**
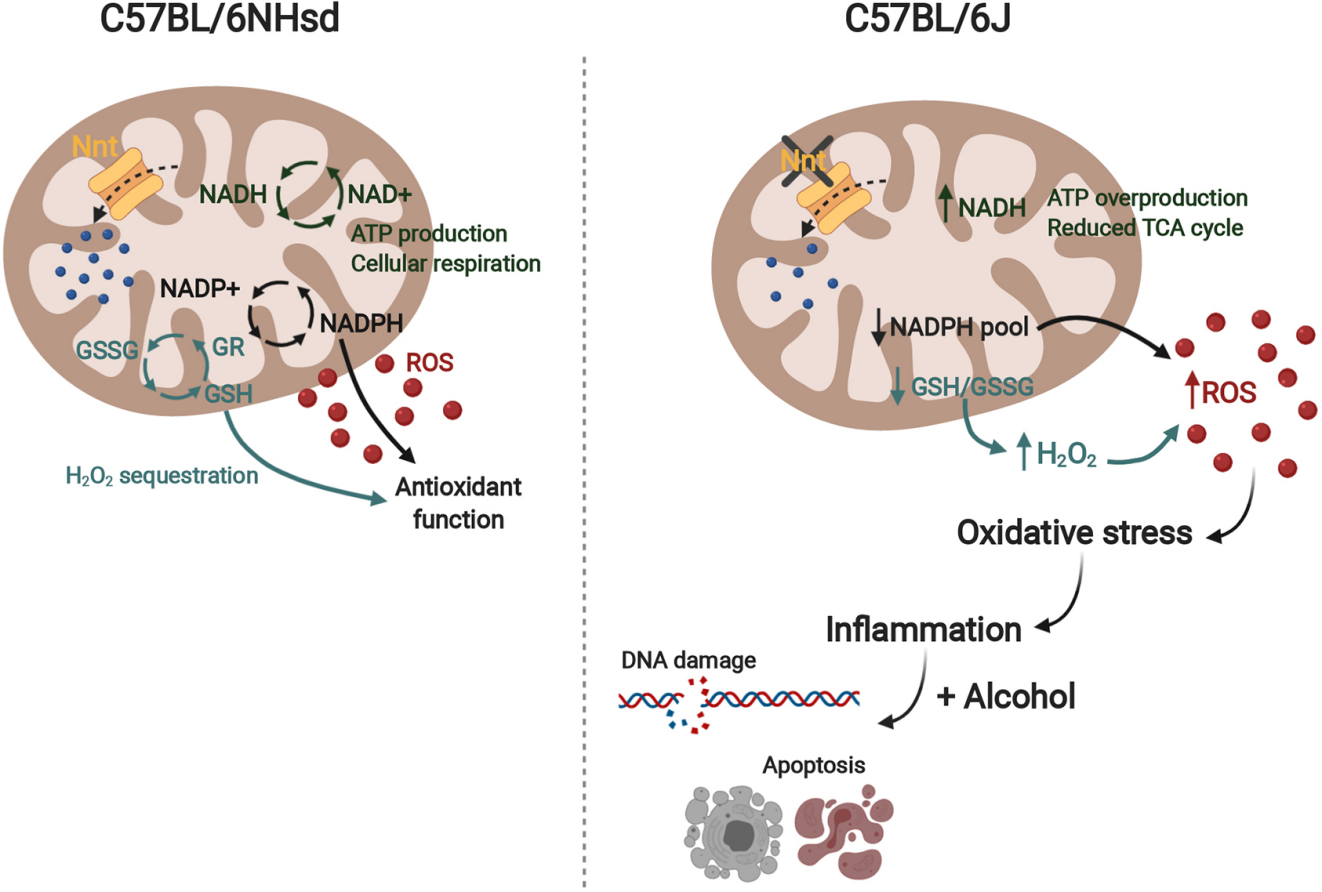
**Schematic representing a hypothetical mechanism contributing to differences in alcohol sensitivity between the 6J and 6N strains.** The *Nnt* mutation in the 6J strain could affect reactive oxygen species (ROS) breakdown in the mitochondria, leading to higher baseline oxidative stress and inflammation. In the presence of alcohol, 6J mice would undergo increased apoptosis and DNA damage, ultimately resulting in more severe craniofacial and CNS anomalies. GR, glutathione reductase; GSSG, glutathione disulfide; GSH, glutathione; NAD+/NADH, nicotinamide adenine dinucleotide (+ hydrogen); NADP+/NADPH, nicotinamide adenine dinucleotide phosphate; Nnt, nicotinamide nucleotide transhydrogenase.

The most well-studied difference between the 6J and 6N strains is the *Nnt* mutation. Nnt is a component of the mitochondrial inner membrane that passes hydrogen atoms that are then used in the conversion of NADP+ to NADPH, an important co-enzyme that regulates metabolism along with NADH. A primary function of NADPH is the removal of ROS from the mitochondria (Fig. 7). NADH is the reduced form of NAD+, and these co-factors are important for redox metabolism, cellular respiration and ATP production. In addition, NADPH converts glutathione from the oxidized (GSSG) to the reduced (GSH) state via glutathione reductase (GR). GSH neutralizes ROS and sequesters and eliminates H_2_O_2_. Reductions in Nnt disrupt the NADPH/NADH balance, causing smaller NADPH pools and lower GSSG/GSH conversion, with less capacity for ROS removal, as well as increased NADH, leading to an overproduction of ATP and dysregulation of glycolysis and the tricarboxylic acid cycle. Although little research has been done on the effect of the *Nnt* mutation on ROS levels in the 6J embryo, endothelial cells from 6J mice exhibit increased superoxide production after angiotensin II stimulation and reduced glutathione peroxidase activity compared to 6N mice (both substrains obtained from The Jackson Laboratory), indicating altered mitochondrial function as a result of the *Nnt* mutation (Leskov et al., 2017). In addition, the *Nnt* mutation has been shown to be a modifier of other genetic mutations, such as *Bcl2l2* (Navarro et al., 2012) and mitochondrial superoxide dismutase (Huang et al., 2006). Increased DNA damage and altered immune signaling have been observed in the 6J strain compared to others in response to other chemical and environmental stressors, including in the lung after exposure to 1,3-Butadiene, a carcinogenic inhalant (Chappell et al., 2017), and in the brain following postnatal hypoxic ischemia (Wolf et al., 2016), although the specific effects seem to be exposure, organ and age dependent. Aberrant Nnt function has also been implicated in cancer, indicating a possible role in cell growth (Ho et al., 2017). A build-up of ROS as a result of the *Nnt* mutation could predispose the embryo to be sensitive to external stressors such as alcohol exposure, which produces oxidative stress on its own (Brocardo et al., 2011; Henderson et al., 1995, 1999).

Oxidative stress can induce inflammation and expression of pro-apoptotic molecules NF-κB and p53 and oxidative stress proteins HIF-1α and PPAR-γ (Reuter et al., 2010). The *Nnt* mutation has also been directly linked to increased expression of HIF-1α in the mouse liver. This molecule is critical for cellular response to hypoxia and can protect against oxidative stress. While *Hif1a* expression did not differ between the strains at baseline, it was downregulated by PAE in the 6J strain at the E7.5 time point. The current study found that the 6J strain had increased expression of genes related to inflammation at baseline. The 6N mice showed an upregulation of inflammation-related genes at E7.5, whereas the 6J mice did not show many alcohol-induced changes in inflammatory pathways at either time point, possibly due to the fact that immune signaling genes were already comparatively activated in the 6J mice at baseline. Interpretation of the upregulation of inflammatory signaling in the 6J relative to 6N strain at E7.0 is limited because the exact function of immune molecules during gastrulation remains under investigation. Early macrophages are detected in the yolk sac during neurulation (∼E9 in mice) (Naito, 2008), whereas cells from a neutrophil lineage do not emerge in the embryo until E11.5 (McGrath et al., 2014), far after the time points under observation here. However, cytokines and chemokines have been suggested to play a role in cell migration (Nair and Schilling, 2008; Katsumoto and Kume, 2011), cell adhesion and tissue remodeling during gastrulation (Aller et al., 2014). In addition, it is possible that some of the pro-inflammatory signals are due to transfer from maternal circulation or the placenta. A direct link between the timing of immune signaling activity (higher at baseline in the 6J mice versus PAE-induced activation in the 6N mice) and differences in alcohol sensitivity between the strains remains to be determined.

Motile and immotile cilia play important roles throughout embryonic development. Previous work from our laboratory has demonstrated that alcohol administered during neurulation alters over 100 cilia genes in the neural tube within the first 6 h after exposure (Boschen et al., 2020). During gastrulation, motile cilia in the primitive node beat to create a morphogenic gradient that regulates left–right asymmetry. Previously, we have shown that knockdown of the cilia gene *Mns1* results in increased incidence and severity of ocular and craniofacial defects following gastrulation-stage alcohol exposure (Boschen et al., 2018), indicating a possible role for cilia dysfunction in the development of prenatal alcohol-related birth defects. Cilia-related genes in the current dataset were identified through comparison of each gene list with the CiliaCarta compendium (Van Dam et al., 2019). Gastrulation-stage alcohol exposure altered cilia-related genes in both strains but to a greater degree in the 6J strain (25 cilia genes in the 6J mice versus 21 genes in the 6N mice at E7.25; 101 cilia genes in the 6J mice versus 24 genes in the 6N mice at E7.5; Table S9). Immotile cilia, called primary cilia, are responsible for transduction of the Shh pathway, as Smo is trafficked into the cilia following binding of Shh to Ptch1, and the Gli transcription factors are processed within the cilia axoneme. In the 6J mice, multiple genes within the Shh pathway (*Shh*, *Ptch1*, *Smo*, *Gli3*) were downregulated 12 h after alcohol. This time point also coincided with a relatively large increase in the number of cilia genes dysregulated by alcohol in this strain compared to the 6N strain. Further investigation is needed to determine whether the cilia genes altered by alcohol exposure in the 6J strain are directly related to the downregulation of Shh pathway genes or are indicative of any significant motile or immotile cilia dysfunction.

The 6J and 6N strains are widely used to study the effects of prenatal drug exposure. Factors such as timing of alcohol exposure (gastrulation versus neurulation), time elapsed between alcohol administration and tissue collection [e.g. 3 h in Green et al. (2007), 6-12 h in our study], and specific tissue type assessed (head fold tissue versus whole embryo) contribute to the differences between previously published gene expression profiles and those reported here. Our previous work (Boschen et al., 2020) sequenced RNA collected from rostroventral neural tube tissue 12 h or 24 h after alcohol in the 6J strain only. Despite methodological differences between these experiments, common targets of alcohol are apparent when the studies are compared. Mitochondrial function and ribosome biogenesis have been reported to be disrupted in multiple models of FASD (Green et al., 2007; Garic et al., 2014; Boschen et al., 2020; Berres et al., 2017) and identified as downregulated pathways in the 6J PAE-treated embryos at E7.5 in the current study. Compromised ribosome biogenesis and mitochondrial function could be indicative of impaired cell growth as synthesis of ribosomes is necessary for cell cycle progression. Cell motility and adhesion have also been determined to be targets of alcohol during early gestation (Dou et al., 2013; Boschen et al., 2020; Green et al., 2007). Cell motility was upregulated in the 6J versus 6N mice at E7.0 in the current study; however, pathways related to cell movement were not enriched by PAE at either time point. Finally, competition between alcohol and retinoic acid (RA) as a mechanism of prenatal alcohol pathogenesis has been a long-standing hypothesis in the field (Deltour et al., 1996; Johnson et al., 2007). Although the current study did not find statistical enrichment of any RA pathways, three genes related to RA signaling were dysregulated after PAE: lecithin retinol acyltransferase (*Lrat*; +0.42 Log2FC in 6J mice at E7.25), retinoic acid receptor-α (*Rara*; +0.46 Log2FC in 6N mice at E7.25) and cellular retinoic acid binding protein 2 (*Crabp2*; −1.04 and −0.87 Log2FC in 6J and 6N mice, respectively, at E7.5). However, interpretation of these single genes is difficult in the absence of other changes to the pathway. RA has been shown to be a regulator of Shh signaling (Ribes et al., 2006; Helms et al., 1997), which was significantly downregulated in the 6J strain 12 h after PAE, although it is beyond the scope of this study to determine whether this change was related to RA signaling.

These data provide information about gene expression patterns in two widely used strains of mice across normal gastrulation and in response to a teratogen. The web tool created to allow for exploration of the dataset visually demonstrates the dynamic nature of certain genes across gastrulation (e.g. *Shh* increases expression over time, *Fgf5* shows reduced expression). The tool will also provide a valuable resource during experimental design, as there are significant differences in gene expression between the two strains that might support the use of one over the other for certain paradigms. The future directions of this study will explore the nuances of gene expression profiles in these two strains, including whether biological sex contributes to prenatal alcohol sensitivity. While all time points used in this study occur prior to gonadal sexual differentiation, differences in gene expression and growth rates have been reported between male and female pre-implantation embryos (Deegan et al., 2019; Werner et al., 2017). Although no sex differences were apparent in the differentially expressed genes in this study, as determined by the consistency between samples (Figs S1-S7), this question needs to be fully explored. In addition, our study used whole embryo tissue, whereas newer sequencing technologies such as single-cell and spatial transcriptomics will allow for investigation of localized mRNA expression patterns, spatiotemporal cell–cell interactions, and a direct link between gene expression and tissue morphology in the gastrulating embryo.

In conclusion, our study demonstrates that a pre-existing genetic susceptibility can mediate sensitivity to teratogens such as alcohol in mice. Not only did the sensitive 6J mice show a larger response to PAE in sheer number of genes/biological pathways affected, but pathways regulating cell death, proliferation, and craniofacial and CNS development were altered to a greater degree in this strain. We hypothesize that the known mutation in *Nnt* in the 6J strain predisposes these embryos to have increased expression of inflammatory signaling genes than make them more sensitive to the addition of an external stressor such as PAE (Fig. 7). Understanding how genetic variability can mediate risk and resiliency to PAE can help elucidate how alcohol acts on the embryo at the cellular level and, ultimately, assist in identifying candidate genes as biomarkers of PAE in the human population.

## MATERIALS AND METHODS

### Animals

Male and female adult C57BL/6J (The Jackson Laboratory, Bar Harbor, ME, USA; Stock #000664) and C57BL/6NHsd (Envigo, Indianapolis, IN, USA) mice (*Mus musculus*) were obtained. Males were housed singly and females were housed in groups up to five in standard polycarbonate cages with cob bedding, shelter and nesting material. Mice had *ad libitum* access to food (Prolab Isopro RMH 3000, LabDiet, St Louis, MO, USA) and water and were maintained on a 12:12 h light/dark cycle. Up to two female mice were placed into the cage of a male for each 2 h mating session. Upon discovery of a vaginal plug, E0 was defined as the beginning of the mating session (Fig. 1). All experimental procedures were approved by the Animal Care and Use Committee at The University of North Carolina at Chapel Hill (UNC) and were performed in accordance with NIH Guidelines (Approval #18-203). On E7.0, dams were weighed and pregnant dams were either dissected immediately or assigned to one of the experimental treatment groups.

### Alcohol exposure paradigm (PAE)

On E7.0, dams were administered two doses of 2.9 g/kg ethanol (25% vol/vol; Pharmaco-Aaper, Brookfield, CT, USA) in Lactated Ringer's solution 4 h apart via intraperitoneal injection (Fig. 1). This dose and pattern of alcohol exposure results in maternal blood alcohol concentrations of ∼400 mg/dl (O'Leary-Moore et al., 2010). Control mice were administered an equal volume of Lactated Ringer's solution (1.5 ml/100 g body weight).

### RNA isolation

RNA was collected from embryos either before alcohol administration (E7.0) or 6 h or 12 h after the first alcohol injection (E7.25 or E7.5) (Fig. 1). Dams were sacrificed via CO_2_ followed by cervical dislocation, and embryos were dissected from the placenta. All extraembryonic tissue was removed and embryos were stage matched based on morphological assessment (Theiler Stages 10-11; representative image in Fig. 2A). A total of six embryos per group were used, with no more than two embryos collected per litter to minimize litter effects. Sex was not considered as a biological variable as all time points occur prior to gonadal sexual differentiation. RNA was isolated using an RNeasy Plus Micro Kit (Qiagen, Germantown, MD, USA), and RNA concentrations and purity were assessed using a NanoDrop 2000 and Qubit 3.0 Fluorimeter (Thermo Fisher Scientific, Waltham, MA, USA). A separate group of samples was collected at the E7.0 time point and isolated for validation of gene expression using quantitative reverse transcription PCR (qRT-PCR). Expression of *Wdfy1*, *Entpd4* and *Efcab7* was analyzed in each strain and found to validate the RNA-seq results from this time point (Fig. S9). All samples were run in triplicate (*n*=6/strain).

### RNA-seq

A total of six samples per group were submitted for sequencing. Libraries for RNA-seq were prepared using the SMARTr Ultra Low Input RNA (Clontech, Mountainview, CA, USA) and Nextera XT DNA (Illumina, San Diego, CA, USA) kits by the UNC High-Throughput Sequencing Facility. Samples were pooled only for sequencing, after RNA extraction and library preparation. For E7.0 samples (12 embryos total), there were four samples per pool (two/group), three pools total, one pool per lane. For E7.25 and E7.5 samples (24 samples/time point), there were four samples per poll (one/group), six pools total, one pool per lane. Paired-end (50 bp) sequencing was performed (Illumina HiSeq 4000).

### RNA-seq and qRT-PCR data analysis and display

Reads were filtered and aligned as described previously (Boschen et al., 2020). Transcript abundance was measured using Salmon (Patro et al., 2017), and differential expression tests were performed using DESeq2 1.22.2 (Anders and Huber, 2010). Gene expression differences were considered significant at an adjusted *P*-value threshold of 0.05. At the E7.5 time point, three outliers were detected and removed from the analysis: one from the 6J vehicle-treated group and two from the 6N PAE group. Final sample sizes are noted in the figure captions. We used gProfiler 0.1.6 (Raudvere et al., 2019) to detect significantly enriched pathways among differentially expressed genes, primarily using Gene Ontology (GO) (Ashburner et al., 2000; The Gene Ontology Consortium, 2018), the Kyoto Encyclopedia of Genes and Genomes (KEGG) (Kanehisa et al., 2019; Kanehisa and Goto, 2000), Reactome (Jassal et al., 2020; Fabregat et al., 2018) and HPO (Köhler et al., 2019). In addition, differentially expressed genes were assayed in a *de novo* network analysis using Ingenuity Software (Qiagen). For the E7.0 time point, network analysis was limited to 35 molecules (genes/proteins/protein complexes) per network due to the small number of input genes. For the E7.25 and E7.5 time points, analysis was limited to 70 focus molecules per network. Networks were ranked by the −log_10_ Fisher's exact *P*-value testing the likelihood of a similar network being formed by the same number (35 or 70) random molecules. Gene lists were also compared to the CiliaCarta compendium (Van Dam et al., 2019) to analyze the number of cilia-related genes disrupted by alcohol at each time point and in each strain.

qRT-PCR data were analyzed with unpaired Student's *t*-tests corrected for multiple comparisons and *P*<0.05 was designated as statistically significant.

The gene expression data browser web tool was developed using the R shiny framework hosted through the Apache HTTP webserver. Several packages are used to process and display the gene expression data, including the tidyverse, here, ggplot2, reactable, dqshiny and shinylogs packages. The computer code for the data browser is available through github (https://github.com/mbergins/Embryo-Genes).

## Supplementary Material

Supplementary information
