# Supplementary material for: Transcriptomic analyses of gastrulation-stage mouse embryos with differential susceptibility to alcohol
Source: Dis Model Mech. 2021 Jun 17;14(6):dmm049012. doi: 10.1242/dmm.049012 (PMC8246266; doi:10.1242/dmm.049012)
Supplement: Supplementary information [file dmm-14-049012-s1.pdf]

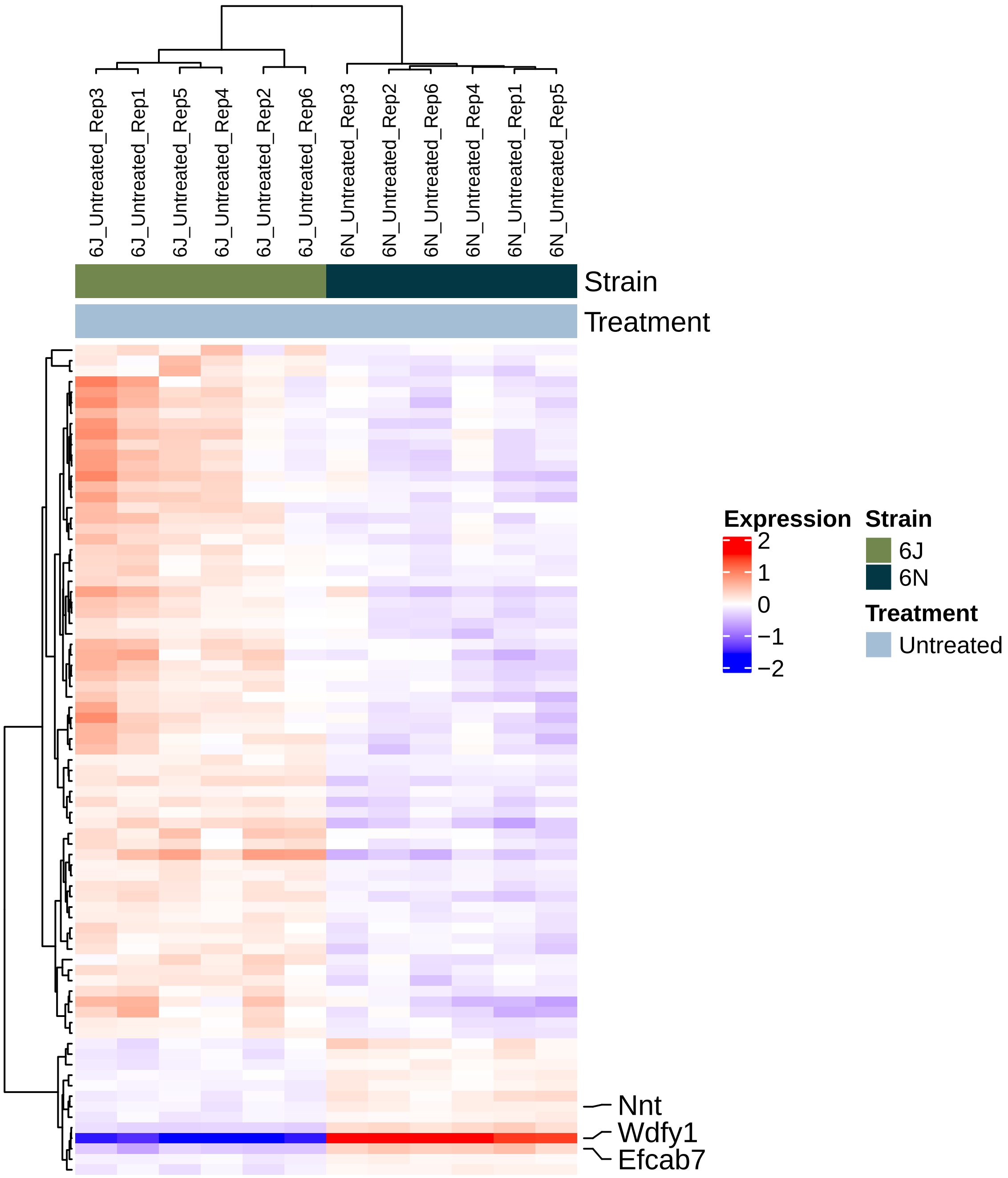

Fig. S1. Median-centered VST-normalized expression values for all differentially expressed genes for each replicate in the E7.0 baseline analysis. n = 6 replicates/group. Gene expression was median centered and clustered hierarchically using 1-Pearson correlation distance. Red = higher median-centered expression, blue = lower median-centered expression.

Disease Models & Mechanisms • Supplementary information

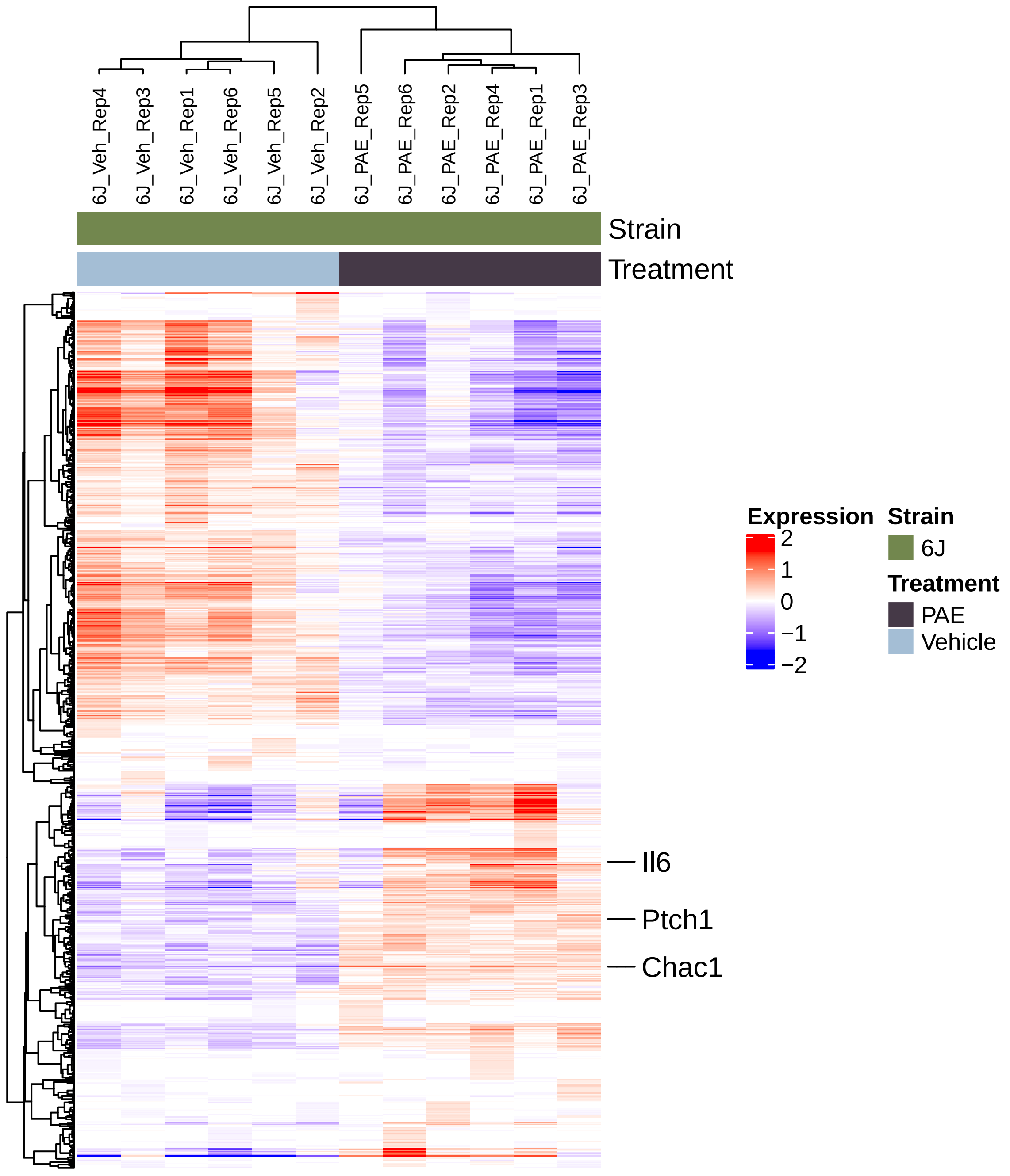

Fig. S2. Median-centered VST-normalized expression values for all differentially expressed genes for each replicate in the vehicle-treated 6N and 6J analyses at E7.25. Significant genes from the E7.25 time point. n = 6 replicates/group. Gene expression was median centered and clustered hierarchically using 1-Pearson correlation distance. Red = higher median-centered expression, blue = lower median-centered expression.

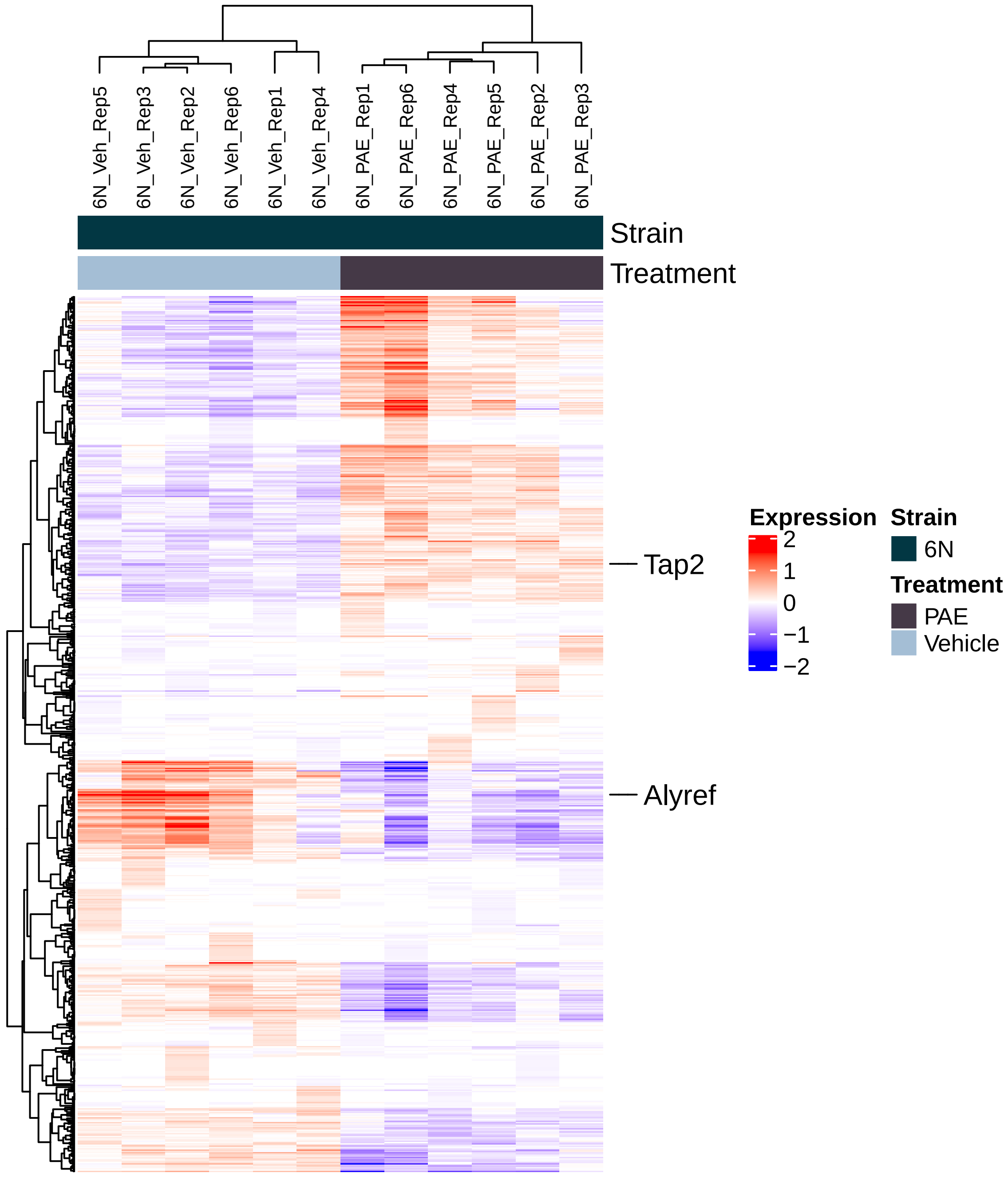

Fig. S3. Median-centered VST-normalized expression values for all differentially expressed genes for each replicate in the vehicle-treated 6N and 6J analyses at E7.5. Significant genes from the E7.5 time point. n = 6 replicates in the 6N group, 5 replicates for the 6J group. Gene expression was median centered and clustered hierarchically using 1-Pearson correlation distance. Red = higher median-centered expression, blue = lower median-centered expression.

Disease Models & Mechanisms • Supplementary information

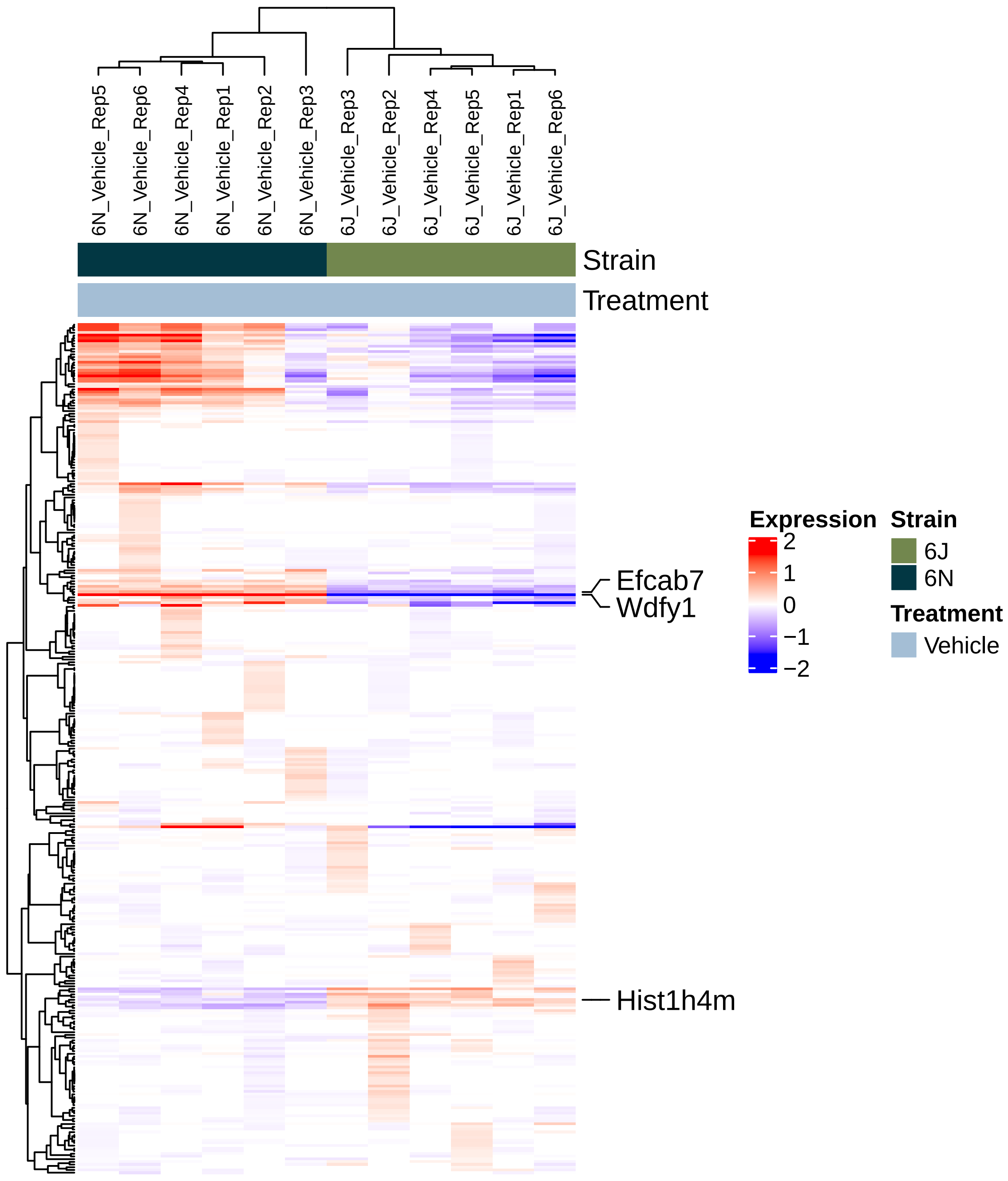

Fig. S4. Median-centered VST-normalized expression values for all differentially expressed genes for each replicate in PAE vs. Vehicle-treated 6J's at the E7.25 time point. Significant genes from PAE vs. vehicle-treated 6J's. n = 6 replicates/group. Gene expression was median centered and clustered hierarchically using 1-Pearson correlation distance. Red = higher median-centered expression, blue = lower median-centered expression.

Disease Models & Mechanisms • Supplementary information

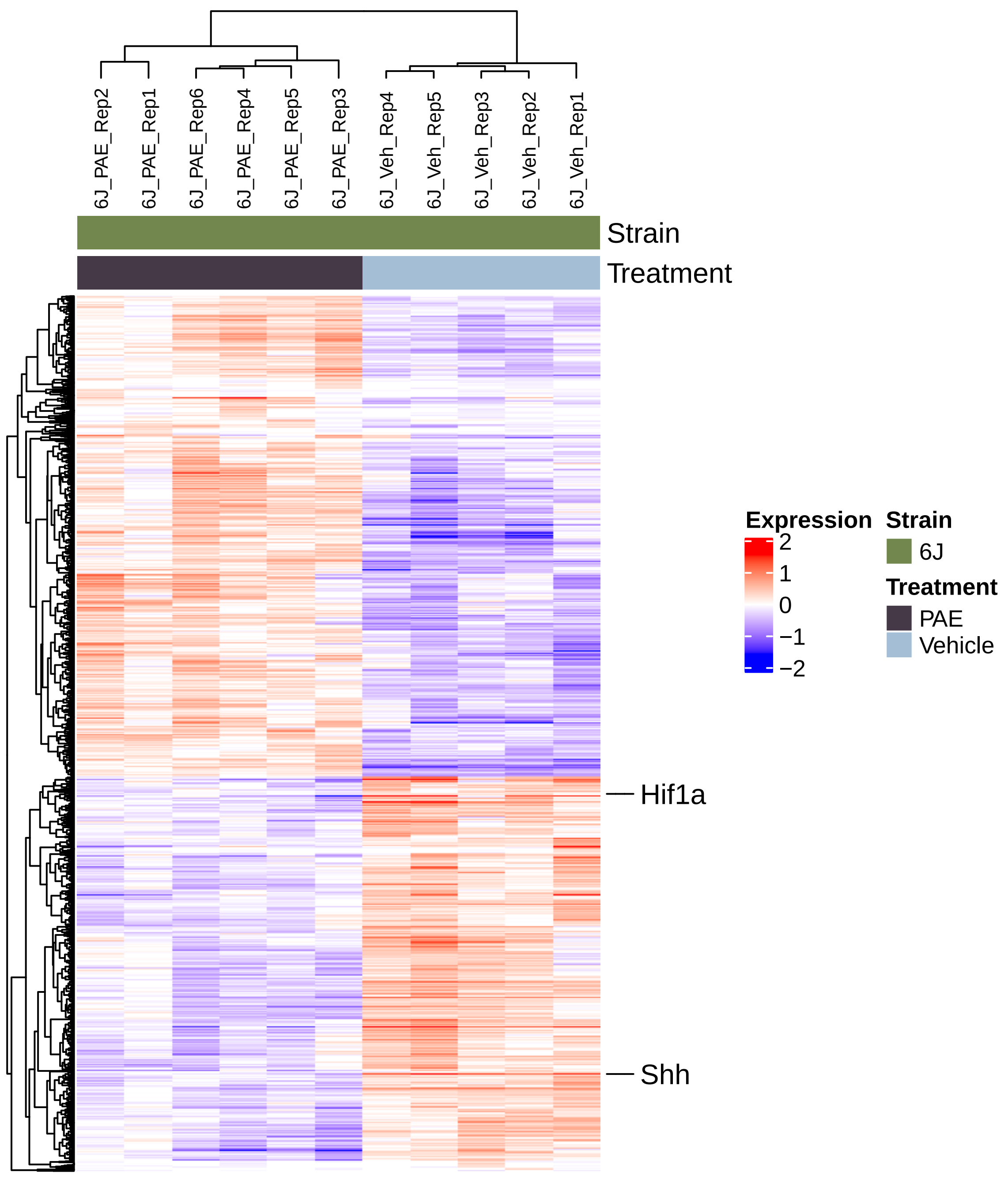

Fig. S5. Median-centered VST-normalized expression values for all differentially expressed genes for each replicate in PAE vs. Vehicle-treated 6N's at the E7.25 time point. A) Significant genes from PAE vs. vehicle-treated 6N's. n = 6 replicates/ group. Gene expression was median centered and clustered hierarchically using 1-Pearson correlation distance. Red = higher median-centered expression, blue = lower median-centered expression.

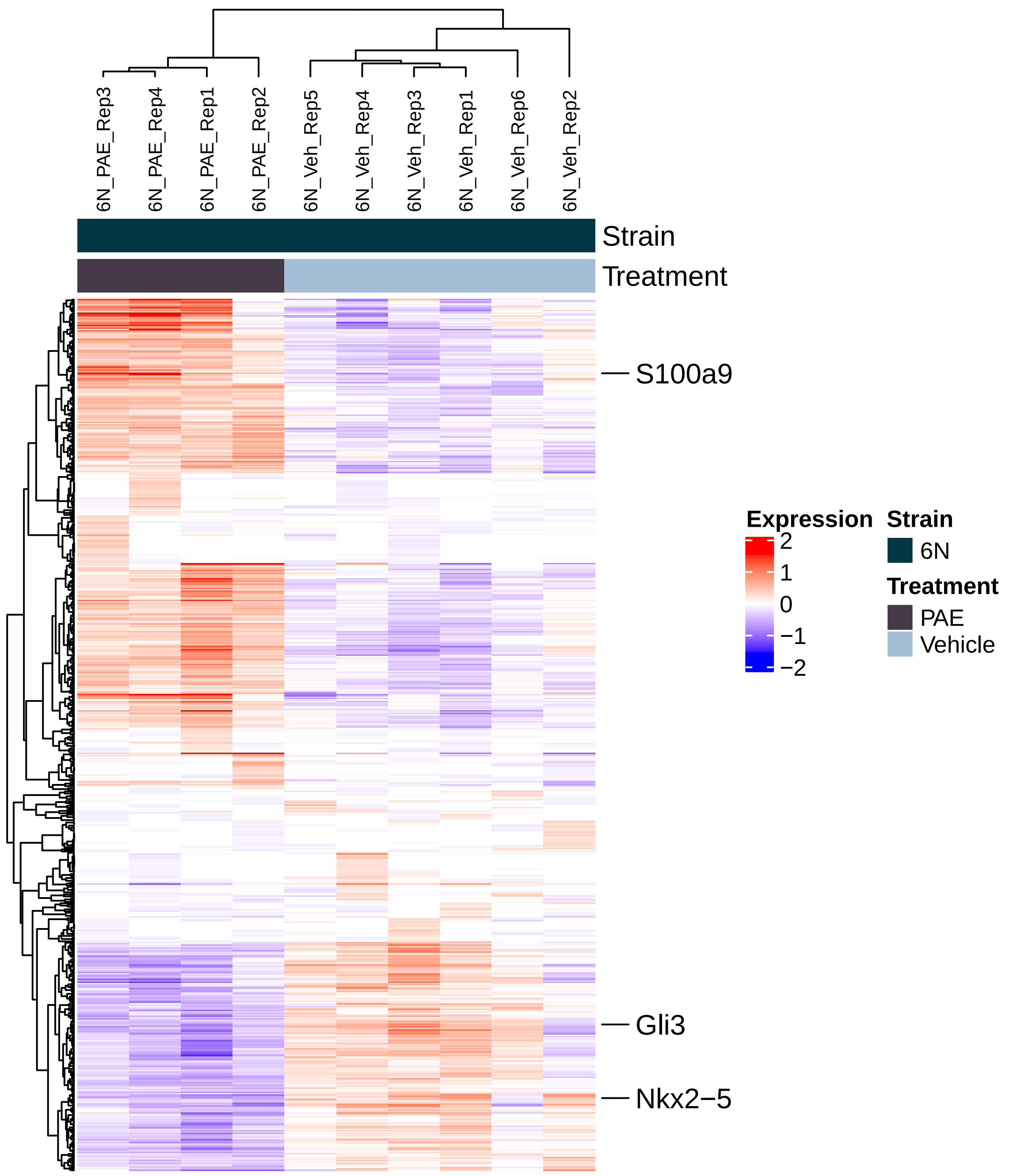

Fig. S6. Median-centered VST-normalized expression values for all differentially expressed genes for each replicate in PAE vs. Vehicle-treated 6J's at the E7.5 time point. Significant genes from PAE vs. vehicle-treated 6J's. n = 5 replicates for the vehicle-treated group, 6 replicates for the PAE group. Genes and samples were hierarchically clustered by 1-Pearson correlation distance. Red = higher median-centered expression, blue = lower median-centered expression.

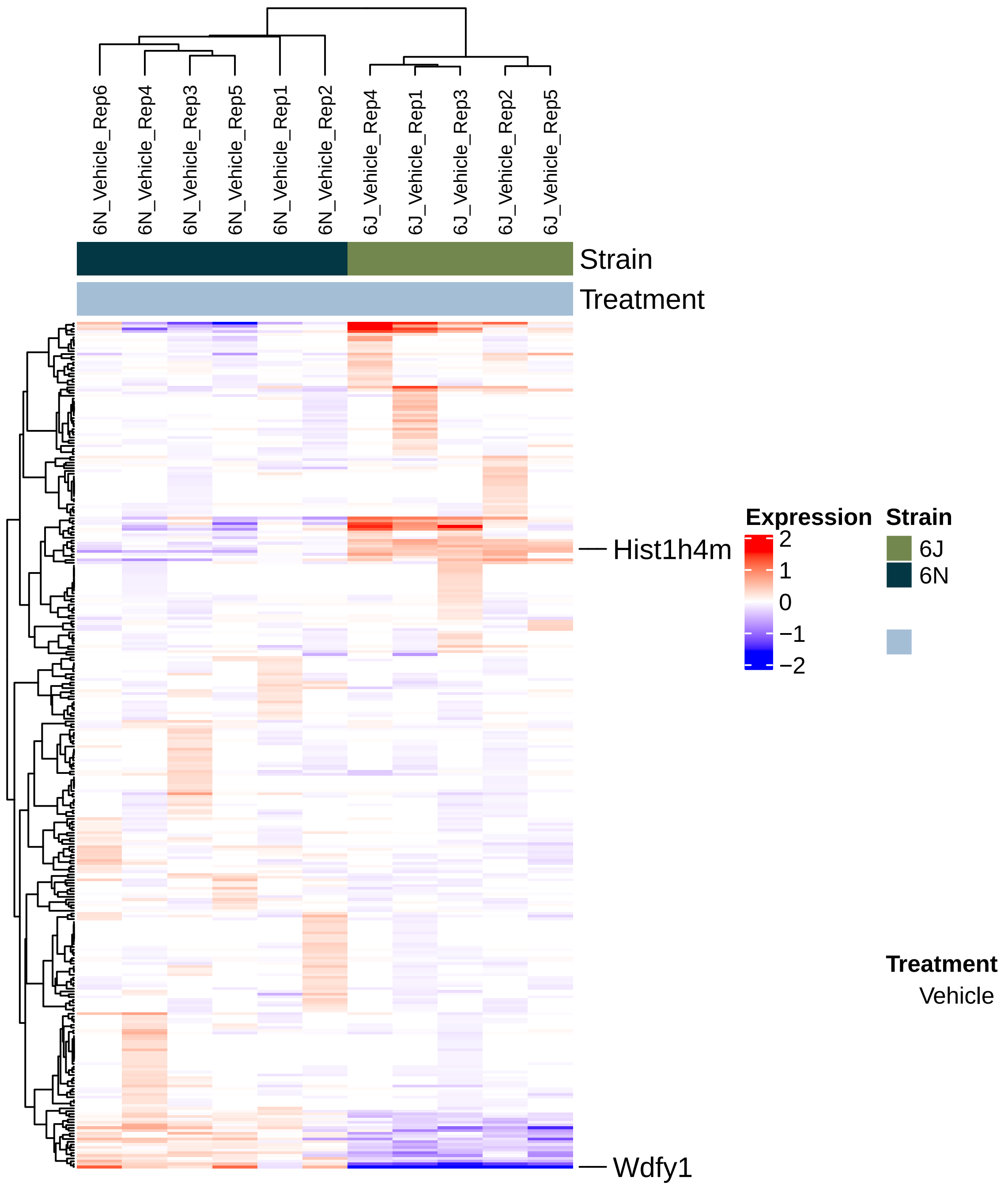

Fig. S7. Median-centered VST-normalized expression values for all differentially expressed genes for each replicate in PAE vs. Vehicle-treated 6N's at the E7.5 time point. Significant genes from PAE vs. vehicle-treated 6N's. n = 6 replicates in the 6N group, 4 replicates for the 6J group. Genes and samples were hierarchically clustered by 1-Pearson correlation distance. Red = higher median-centered expression, blue = lower median-centered expression.

Disease Models & Mechanisms • Supplementary information

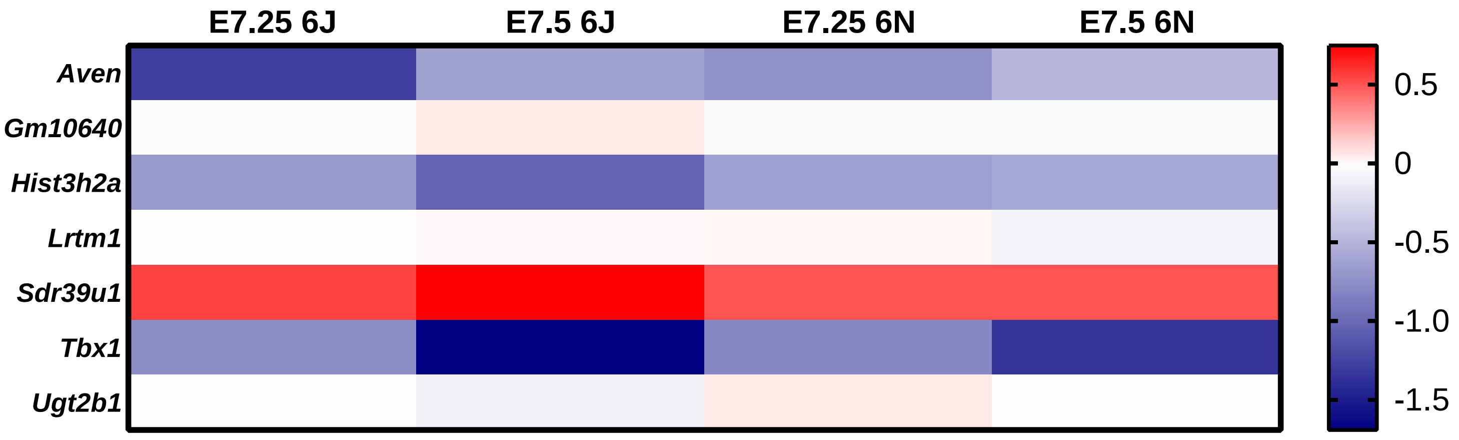

Fig. S8. Seven genes differentially regulated by PAE in both strains at both time points. Data are shown as the Log2FC for each PAE group vs. the appropriate vehicle-treated group from that strain and time point. Red = up-regulated genes, blue = downregulated genes.

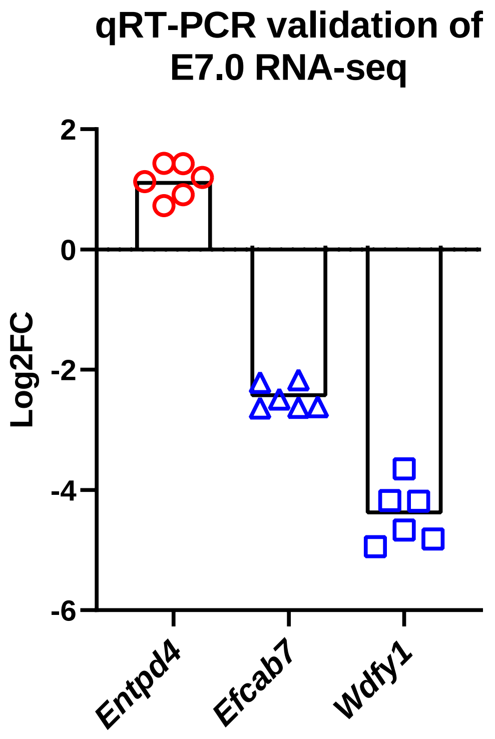

Fig. S9. Validation of three differentially expressed genes at the E7.0 time point. qRT-PCR was performed on different samples than those used for RNA-seq (n = 6/strain) to assess expression of *Entpd4, Efcab7*, and *Wdfy1*. All genes were expressed at significantly different levels in the 6J vs. 6N in the direction expected based on the RNA-seq output: *Entpd4:* +1.14 Log2FC in 6J vs. 6N, $t(10) = 4.473$, $p = 0.0012$; *Efcab7*: -2.46 Log2FC in 6J vs. 6N, $t(10) = 14.36$, $p < 0.0001$; *Wdfy1:* -4.41 Log2FC in 6J vs. 6N, $t(10) = 20.05$, $p < 0.0001$. Error bars indicate standard error of the mean.

Disease Models & Mechanisms • Supplementary information

Table S1. **Selected gProfiler results from differentially regulated genes between the 6J and 6N strain at baseline (E7.0).** Pathways associated with down-and up-regulated genes are listed separately.

Up-regulated Genes

Gene Ontology: Biological Process

Term name	Term ID	# of genes	Log10 *p*-value
Myeloid leukocyte migration	GO:0097529	8	3.68
Cytokine production	GO:0001816	12	2.71
Defense response	GO:0006952	17	2.58
Response to external stimulus	GO:0009605	22	2.22
Inflammatory response	GO:0006954	11	2.15
Cell migration	GO:0016477	15	1.94
Regulation of immune system process	GO:0002682	15	1.94
Cytokine-mediated signaling pathway	GO:0019221	8	1.49
Cell motility	GO:0048870	15	1.44
Localization of cell	GO:0051674	15.00	1.44

Gene Ontology: Molecular Function

Term name	Term ID	# of genes	Log10 *p*-value
Cytokine binding	GO:0019955	7.00	4.45
ATPase activity, coupled	GO:0042623	6.00	1.32

KEGG: Biological Process

Term name	Term ID	# of genes	Log10 *p*-value
Cytokine-cytokine receptor interaction	KEGG:04060	7.00	2.05

Table S2. **Top ten de novo gene network lists for all time points.** A) E 7.06J vs. 6N (before alcohol), B) E7.25 vehicle-treated 6J vs. 6N, C) E7.5 vehicle-treated 6J vs. 6N, D) E7.25 6J Alcohol vs. Vehicle, E) E7.25 6N Alcohol vs. Vehicle, F) E7.5 6J Alcohol vs. Vehicle, G) E7.5 6N Alcohol vs. Vehicle. Gene networks created with Ingenuity Pathway Analysis.

A **E 7.0 6J vs. 6N**

	Diseases and Functions	-log(Fisher's exact *p*-value)	Molecules in Network	Molecules
1	– Cell Cycle<br>– Drug Metabolism<br>– Molecular Transport	31	15	Aak1, Acad11, Adck2, Ahnak, App, Arfgap3, Arglu1, Atp8b1, Ccdc88c, Clpb, Dnah14, Dnajc28, Dynlrb2, Dynlt1, Hspa9, Hspb9, Igsf6, Klf12, Lilra5, Mbnl3, Mmp3, Myc, Myo1d, Neat1, Nnt, Nr1h4, Rxr, Sez6l, Sirt5, Tex2, Tktl2, Tmem267, Tnf, Tretinoin, Vgll3
2	– Cancer<br>– Immunological Disease<br>– Inflammatory Disease	28	14	26s Proteasome, Anxa11, Btaf1, Cd3, Creb, Cx3cr1, Efcab7, Entpd4, Erk, Fsh, Fxyd5, Gpcr, Histone H3, Hsp70, Htr2b, Ide, Insulin, Mapk, Nfkb (Complex), Pdgf Bb, Pi3k (Complex), Pka, Pkc(S), Plc, Proinsulin, Ras Homolog, Sct, Slc25a12, Src (Family), Tiam1, Tpm4, Ubiquitin, Uqcc2, Vegf, Wt1
3	– Cellular Movement<br>– Immune Cell Trafficking<br>– Inflammatory Response	25	13	Ap1, Ccl4, Collagen Alpha1, Collagen Type I (Complex), Collagen Type Ii, Collagen Type Iv, Collagen(S), Csf3r, Ddx58, Erk1/2, Fcer1, Fibrinogen, Hsd11b1, Ifn, Ifn Beta, Il-1r, Il1r1, Il1rn, Ldl, Litaf, Mmp, Mmp7, Mmp8, Olr1, Pkc Alpha/Beta, Pro-Inflammatory Cytokine, Rhob, Saa, Sftpd, Sod, Tgf Beta, Tlr, Tnf (Family), Tnfrsf9, Trypsin
4	– Cancer<br>– Endocrine System Disorders<br>– Organismal Injury and Abnormalities	25	13	Akt, Ampk, C1qtnf5, Chemokine, Cytokine, Hp1bp3, Ifi16, Ifn Alpha/Beta, Ifn Type 1, Ifnar, Ige, Igg, Igg1, Igm, Il1, Il12 (Complex), Il12 (Family), Immunoglobulin, Interferon Alpha, Irf7, Kit, Lh, Map2k1/2, Mtdh, N-Cor, Nfkbiz, Nos, Osbpl5, P70 S6k, P85 (Pik3r), Pglyrp1, Pthlh, S100a9, Top2b, Wdfy1
5	– Cellular Movement<br>– Hematological System Development and Function<br>– Immune Cell Trafficking	18	10	Adm2, Atp11b, Ccr1, Cd200, Cdx2, Cx3cr1, D1pas1, Defa1 (Includes Others), Defb104a/Defb104b, Gjb4, Gpr132, Gstp1, Hsh2d, Jnk, Lgals8, Mannan, Mip1, Mir-127, Mrc1, Mrc2, Nipal1, P38 Mapk, Pla2g7, Prr9, Rara, Retnlg, S1pr2, Sparc, Stra6, Tcr, Tlr10, Tmcc3, Tmem184a, Tnfrsf18, Traf5
6	– Cell Morphology<br>– Cellular Assembly and Organization<br>– Post-Translational Modification	16	9	1, 3, 4-Ip3, B4galt4, Brd8, Ceacam10, Ck2, Defb8, E2f8, Egf, Ep400, Epc1, H2az1, H4c4, Hdac2, Hoxc9, Ing2, Ing3, Irs1, Itpkc, Mier2, Mis18a, Nes, Oip5-As1, Papss1, Pcgf2, Pold2, Rbbp4, Rffl, Sap130, Sp3, Sprr2h, St5, Tcf21, Tip60, Tp53, Ube2q2

B **E 7.25 6J vs. 6N Vehicle-treated**

	Diseases and Functions	-log(Fisher's exact *p*-value)	Molecules in Network	Molecules
1	– Antimicrobial Response<br>– Cell Death and Survival<br>– DNA Replication, Recombination, and Repair	39	22	Asb9, Bglap, Casp12, Caspase, Cd200r1, Ces, Ces1b/Ces1c, Ces4a, Cngb3, Cytochrome C, Ecm, Fap, Granzyme, Gzma, Gzmb, Gzmc, Gzmh, Hsp27, Hsp70, Il12 (Family), Il6, Jun/Junb/Jund, Mir124a-1hg, Mt3, Nfkb (Complex), Padi2, Rxr, Serine Protease, Serpinb12, Spink5, Tcr, Tmprss11b, Tmprss7, Trim69, Usp17la (Includes Others)

Disease Models & Mechanisms • Supplementary information

**2**	– Cell-To-Cell Signaling and Interaction<br>– Drug Metabolism<br>– Nervous System Development and Function	34	20	Alpha Catenin, Ampk, Atp1b4, Calpain, Capn11, Cd3, Cd4, Cg, Cpa3, Creb, Efcab7, Fmo3, Fsh, Gabrg2, Glucuronosyltransferase, Hbg2, Histone H3, Hla-A, Hoxd10, Il1, Il1bos, Kcnj1, Mek, Nr2e1, P38 Mapk, Pi3k (Complex), Pkc(S), Ppp1r1b, Psca, Slco2b1, Tm4sf1, Ugt2a3, Ugt2b17, Ugt2b28, Vegf
**3**	– Cell Signaling<br>– Molecular Transport<br>– Vitamin and Mineral Metabolism	32	19	Amylase, Ccl1, Col28a1, Collagen, Collagen Type Ii, Cxcl13, Elastase, Erk1/2, Fcna, Fgf7, Fibrin, Gp6, Hcrt, Hla-Dr, Ifn Alpha/Beta, Il36b, Irak3, Klra7 (Includes Others), Lum, Mhc Class I (Complex), Mhc Ii, Mmp, Ppp1r3a, Pro-Inflammatory Cytokine, Reg3g, Rgs13, Serpina1, Syk/Zap, Tlr, Tnf (Family), Tnfsf15, Trem1, Trypsin, Ucn2, Xcl1
**4**	– Hematological System Development and Function<br>– Hematopoiesis<br>– Humoral Immune Response	30	18	Aim2, Akt, Bcr (Complex), Cd180, Cd36, Ctnna3, Dbh, Fcamr, Gcsam, Gm9573, Gpnmb, Hemoglobin, Ifn Beta, Ifnar, Iga, Igg, Igg1, Igg2a, Igg2b, Igg3, Igm, Il12 (Complex), Insrr, Ly9, Lyg2, Medag, Mhc Class Ii (Complex), Pax5, Plc Gamma, Stat5a/B, Tfap2b, Tnfrsf17, Trim29, Vav, Wap
**5**	– Cell Signaling<br>– Molecular Transport<br>– Vitamin and Mineral Metabolism	25	16	Adcy, Adgrf1, Bdkrb1, Calcrl, Calmodulin, Ccr6, Chemokine, Chemokine Receptor, Collagen Type I (Complex), Cxcr1, Cyp2c9, Dynlt1, Ffar2, Fshr, G Protein, G Protein Alphai, Ghrhr, Gpcr, Grm1, Growth Hormone, Gsk3, Jnk, Lh, Mapk, Myoc, Npy, Pka, Plc, Rac, Ras, Serpina3g (Includes Others), Tacr1, Tsh, Tshr, Voltage-Gated Calcium Channel
**6**	– Cell-To-Cell Signaling and Interaction<br>– Skeletal and Muscular System Development and Function<br>– Visual System Development and Function	23	15	Akr1c3, Aldh1a2, Aldose Reductase, Alp, Ap1, Calml5, Ces1, Collagen(S), Erk, Fcer1, Fgf, Fgf6, Ghrl, Gk, Hdl, Ige, Il23, Krt75, Ldl, Lilrb3, Myh4, Mylk2, Myosin, Nadph Oxidase, Nr1h, Pdgf Bb, Pi3k P85, Prkaa, Prl2c2 (Includes Others), Rap1gds1, Rock, Slfn12l, Src (Family), Tgf Beta, Txk
**7**	– Cardiovascular System Development and Function<br>– Cellular Function and Maintenance<br>– Tissue Morphology	23	15	60s Ribosomal Subunit, Abca13, Adamtsl3, Add1, Alas2, Aldh3b1, Arnt2, Bbox1, Cldn13, Clk1, Ddx54, Ess2, Fbxl18, Gata2, Gdf2, Grm6, Gypa, Hemgn, Krt40, Ly6a (Includes Others), Mrgprx3, Myc, Nat3, Ncaph, Noc2l, Nsfl1c, Prrg4, Rad21l1, Rffl, Rhag, Rpl39l, Tmem200c, Tsga10ip, Vcp, Wapl
**8**	– Cell Morphology<br>– Cellular Function and Maintenance<br>– Developmental Disorder	21	14	26s Proteasome, 4930417o13rik, Acyl-Coenzyme A, Aim2 Inflammasome, Alas2, Cd209d, Ceacam20, Ctcfl, Cyp1a2, Cytokine, Defb8, Ep400, H4c4, Histone, Histone H4, Hsp90, Ide, Immunoglobulin, Insulin, Interferon Alpha, Klhl38, Midn, Mylip, Paf1, Phosphatidylinositol 3, 4-Diphosphate, Phosphatidylinositol-3-Phosphate, Pla2g2a, Pold2, Ppm1k, Prap1, Proinsulin, Rna Polymerase Ii, Tip60, Tssk1b, Wdfy1
**9**	– Cell-To-Cell Signaling and Interaction<br>– Nervous System Development and Function<br>– Organismal Injury and Abnormalities	21	14	Acot5, Bdnf, Cntnap5, Ctcf, Drd1, Endocannabinoid, Fabp2, Foxa1, Ftsj3, Gpx4, Grb2, H1-1, Kri1, Ly6g6f, Nkx2-5, Olfr503, Or4n2, Pcdhb3, Pcdhb7, Pcdhb8, Plod1, Ppara, Ppp1r1b, Rp1l1, Rps27, S100a10, Scn10a, Scn11a, Sec14l5, Set, Set Complex, Slc17a8, Tespa1, Vgf, Xrn2
**10**	– Cell-To-Cell Signaling and Interaction<br>– Cellular Function and Maintenance<br>– Nervous System Development and Function	21	14	9-Cis-Retinal, Akap5, Apol11b (Includes Others), Apol9a/Apol9b, App, Arglu1, Asic1, Asic2, Asic3, Cdr1, Chrnb3, Crx, Dhx33, Dlg4, Dmrt1, Dmrtc2, Dopamine, Endocannabinoid, Etd, Girk, Gpr37, Gpr78, Hcrtr1, Ifn, Ifnar1, Irak, Myot, Opn5, Rho, Rnu12, Sectm1, Snrnp, Spata16, Tlr7, Trpv3

C E 7.5 6J vs. 6N Vehicle-treated

	Diseases and Functions	-log(Fisher's exact *p*-value)	Molecules in Network	Molecules
**1**	– Drug Metabolism<br>– Lipid Metabolism<br>– Small Molecule Biochemistry	50	26	Aim2, Amy2a, Amylase, Anxa13, Bglap, Cd200r1, Cdh16, Cebpe, Ces, Ces1, Ces3, Ces4a, Collagen Type Ii, Ctrb2, Cyp7a1, Erk1/2, Fgf7, Growth Hormone, Hcrt, Il36g, Klrc1, Magea3 (Includes Others), Mug1/Mug2, Mycs, Nxph2, Prex1, Proinsulin, Pxr Ligand-Pxr-Retinoic Acid-Rxrα, Rxr, Slco1b3, Syt2, T3-Tr-Rxr, Tph2, Ubash3a, Ucn3
**2**	– Connective Tissue Development and Function<br>– Digestive System Development and Function<br>– Skeletal and Muscular System Development and Function	42	23	Ackr2, Afm, Akt, C3ar1, Cd207, Cd55, Collagen Type I (Complex), Collagen Type Iv, Collagen(S), Csta, Ctsk, F9, Fhit, Fmod, Gm9573, Hephl1, Iga, Igg2a, Igg2b, Il23, Itga11, Ldl, Lyg2, Mucin, Myoc, Or51e2, Pdgf Bb, Prg3, Reg3a, Scgb3a1, Serpinb7, Siglec15, Slc13a1, Tcl1a, Tgf Beta
**3**	– Post-Translational Modification<br>– Protein Degradation<br>– Protein Synthesis	42	23	Ano3, Ccl1, Ccl24, Ccl7, Cd209c, Cd8b, Cnga3, Fcer1, Gngt1, Ifn Alpha/Beta, Ifn Beta, Ifnar, Ifnz (Includes Others), Interferon Alpha, Klk12, Klk5, Klra7 (Includes Others), Mhc Class I (Complex), Mhc Class I (Family), Mhc Class Ii (Complex), Mx2, Nfkb (Complex), Pmaip1, Prss42p, Serine Protease, Serpinb10, Slc46a2, Tap2, Tmprss11e, Tmprss15, Tmprss7, Tnf (Family), Tnfrsf17, Trypsin, Usp17la (Includes Others)
**4**	– Cancer<br>– Dermatological Diseases and Conditions<br>– Organismal Injury and Abnormalities	35	20	Adamtsl4, Alb, Atp6v1g3, Bcr (Complex), Chst4, Col8a1, Cox6a2, Creb, Ctnna3, Cxcl3, Cyclin A, Erk, Fhl5, Gabrg2, Gcsam, Gm10408 (Includes Others), Ige, Igg, Igg1, Il1, Il12 (Complex), Il13ra2, Immunoglobulin, Keratin, Krt16, Krt20, Krt73, Lh, Map2k1/2, Pax8, Pck2, Pou2af1, Ppy, Pro-Inflammatory Cytokine, Sos
**5**	– Developmental Disorder<br>– Endocrine System Disorders<br>– Hematological Disease	28	17	26s Proteasome, Asb9, Calmodulin, Cd3, Chemokine, Cxcr6, Cytokine, Efcab7, Gpcr, Gpr119, Gpr22, Gpr37, Histone H3, Histone H4, Hsp70, Hsp90, Itgad, Jnk, Kcnj1, Kcnj16, Micb, Pi3k (Complex), Pka, Pkc(S), Rb, Rho, Slc12a1, Tcr, Tesk2, Tnn, Tp63, Ube2d4, Ubiquitin, Vegf, Wdfy1

6	– Cancer<br>– Endocrine System Disorders<br>– Gastrointestinal Disease	26	16	Abr, Ahctf1, Alms1, Alpk2, Atxn7, Bbox1, Ccdc27, Ccdc88c, Cnnm2, Cntnap3, Ctcf, Ctnnb1, Cyb5b, Fhit, Gabrq, Gjd4, Hepacam, Itprid2, Lhx6, Lmna, Lnx1, Lvrn, Mastl, Nes, Pcdhb8, Pcdhb8, Plec, Rasl11b, Rnf133, Ropn1, Sall1, Spag11b, Ube2i, Ube2q1, Virma
7	– Cancer<br>– Organismal Injury and Abnormalities<br>– Tissue Morphology	23	15	Abcb8, Adam30, Ankrd34c, Atf3, Bpifc, Cpn2, Creb1, Cst9l, Dio2, Dmrt1, Dnm3os, Fbxw21 (Includes Others), Glra1, Hmmr, Ifi202b, Kcnb1, Mapk1, Metalloprotease, Myl6b, Nr1h5, Pdcl2, Prkar2a, Prl3c1, Rest, Rhox3a (Includes Others), Robo1, S6k1, Scn10a, Scn3b, Tp53, Traf6, Trank1, Triiodothyronine, Reverse, Vnn1, Vsig1
8	– Nutritional Disease<br>– Psychological Disorders<br>– Small Molecule Biochemistry	21	14	14, 15-Epoxyeicosatrienoic Acid, Adamdec1, Ar, Beta-Estradiol, Cilp, Cldn18, Cldn6, Drd4, Ear2 (Includes Others), Elovl2, Endothelin, Eppin, Gata2, Gchfr, Gp2, Gpr37, Grk4, Hemgn, Htr6, Ifi202b, Mak, Mhc Class Ii (Complex), Mir-24-3p (And Other Mirnas W/Seed Ggcucag), Msmo1, Myc, Ntf4, Parp8, Pmm2, Pomc, Sprr2g, Sprr2i, Tbx19, Tgm6, Transglutaminase, Zg16
9	– Cell Cycle<br>– Cell Morphology<br>– Cell-To-Cell Signaling and Interaction	19	13	Actl7b, App, Brms1, Ccnb2, Ccnb3, Clec2e/Clec2h, Crebbp, Crybb2, Endothelin, Gjb1, Gng2, Hmgb4, Hspa2, Iqcf5, Kcnn4, Kctd16, Lsmem1, Mac, Mastl, Ms4a15, Nanog, Prkar2a, Psg18 (Includes Others), Ptger2, Rnasel, Sik2, Slc25a13, Stfa2/Stfa2l1, Sycp2l, Tbx22, Tigd4, Ttk, Usp8, Utp, Zdhhc21
10	– Cell Cycle<br>– Cell-To-Cell Signaling and Interaction<br>– Cellular Growth and Proliferation	17	12	9830107b12rik/A530064d06rik, Abcb9, Aim2, Apol11b (Includes Others), Bc048679, Chil3/Chil4, Cntnap5, Crabp1, Ctsk, Cyp4f11, Dyrk2, Eln, Ern2, Esr1, Fmr1, Gchfr, Gvin1 (Includes Others), Hla Class I, Hla-G, Ifi202b, Ifng, Muc2, Nr1d1, Pcdhb14, Pcdhb4, Pla2g7, Rcvrn, Tgfbr2, Th2 Cytokine, Ticam2, Tlr3, Tnfsf9, Tretinoin, Ttc22, Uba6

D **E 7.25 6J Alcohol vs. Vehicle**

	Diseases and Functions	-log(Fisher's exact *p*-value)	Molecules in Network	Molecules
1	– Cancer<br>– Gastrointestinal Disease<br>– Hepatic System Disease	99	64	Aasdh, Abcb8, Aebp2, Aldh3b1, Amfr, Ammecr1, Anxa10, Apbb2, Arhgap23, Atp2a2, Atpase, Bag3, Banp, Bod1, Btbd1, Caap1, Cacybp, Carm1, Carnmt1, Crebrf, Dars1, Eed, Entpd2, Faf1, Fxr2, G3bp1, Glud1, H1-0, Hdac, Hemgn, Hsp70, Hspa5, Hspa8, Hspd1, Hsph1, Klhdc2, Lrrc47, Map1lc3, Mapre1, Mettl17, Mpp6, Mrps27, Noa1, Nr2e1, Ogt, Pcbp1, Pex6, Ppp1r10, Ptov1, Rabl3, Rbm26, Rbpms, Rif1, Rpa, Sfpq, Slc35e1, Slco2b1, Smc4, Spg7, Tmem165, Trim29, Trmt12, Trmt44, Tssk1b, Ube2m, Ubxn2b, Ugt2b17, Vegf, Ywhah, Znf746
2	– Cell Morphology<br>– Drug Metabolism<br>– Endocrine System Development and Function	89	60	Alkbh5, Asb13, B4galt4, Bbc3, Bbs4, Bmt2, Brd7, Brpf3, Cables2, Capns2, Ccnt1, Ccny, Cct3, Cct4, Cdc73, Cnppd1, Cofilin, Ctbp1, Ctdp1, Dazap2, Dnaja1, Eif4a2, Ercc8, Fam91a1, Fkbp5, Gcn5l, Gmcl1, Gtf2e2, Histone, Histone H3, Holo Rna Polymerase Ii, Inpp5k, Ipmk, Kdm1a, Kiaa2013, Mark3, Mbd3, Mcmbp, Mettl3, Mi2, Mlc1, Nars1, Nurd, Oma1, Orc3, P-Tefb, P38 Mapk, P4ha1, Pard3, Pard6b, Parp16, Paxbp1, Per1, Pmpcb, Ppp2r2d, Rhof, Rsrc2, Slc6a12, Snx5, Spata20, Spata3, Srsf2, Srsf9, Stip1, Strn3, Suv39h2, Tada3, Taf10, Taf5, Tip60
3	– Cancer<br>– Cellular Development<br>– Tissue Development	84	58	5730488b01rik, Ahcyl1, Alp, Alpg, Asb15, Bmp1, Cbx6, Chac1, Cirbp, Collagen Alpha1, Commd3-Bmi1, Coq2, Ctbp, Erk, Exog, Exosc6, Farnesyl Transferase, Fgf, Gata6, Get4, Hedgehog, Hes1, Hoxa9, Hoxd10, Leng8, Loxl1, Luc7l, Mafg, Mllt1, Mocs3, Mrps30, Napsa, Nog, Nop56, P3h2, Pip4k2a, Plcd3, Prc2, Prl2c2 (Includes Others), Prmt2, Psca, Ptch1, Rangap1, Riox1, Riox2, Rnr, Rrs1, Sdr39u1, Slfn12l, Smarca5, Sox, Sox17, Sox18, Sox2, Sox4, Spout1, Tcf, Tigd5, Tle1, Trmt10c, Trmt1l, Tsc22d1, Ugcg, Wars2, Wdr77, Wnt, Yars2, Ypel5, Zc3h14, Zmynd19
4	– Cancer<br>– Connective Tissue Disorders<br>– Organismal Injury and Abnormalities	73	53	26s Proteasome, Alyref, Ankrd13a, Apc/Apc2, Arrdc3, Arrdc4, Atp6v0d2, B3gnt5, Barx1, Bmp, Bok, Ccdc155, Cldn5, Clec4g, Cnpy3, Csnk1g3, Ctnna3, Eotaxin, Fam161a, Fgfbp1, Galnt1, Gas1, Gml, Got, Hoxd9, Hsd3b1, Igg, Igg2a, Igg2b, Igll1/Igll5, Il25, Irak3, Lman2l, Lrig1, Lrrtm1, Mhc Class I (Family), Mhc Ii, Mir101, Mrm3, Msl2, Mto1, Nfkb (Complex), Noct, Otulin, P Glycoprotein, Rab6b, Rbm15b, Rnf11, Rnf149, Slc22a16, Srxn1, Tespa1, Tmem245, Tnfrsf17, Tnfsf15, Tpcn2, Trim10, Trim44, Trim8, Tspan33, Tysnd1, Ube2, Ube2q2, Ube2r2, Ubiquitin, Ubiquitin Ligase, Vacuolar H Atpase, Vash1, Wipi1, Zfand5
5	– Cardiac Necrosis/Cell Death<br>– Cardiovascular Disease<br>– Cell Death and Survival	60	47	Adrb, Alcohol Group Acceptor Phosphotransferase, Alpha Actin, Asb9, Becn1, Btg3, Cab39, Calcineurin A, Cdc25b, Cg, Chrna6, Chrnb3, Cited2, Creatine Kinase, Dapk3, Dll3, Dusp10, E2f1, Eno2, Ephb, Etfbkmt, Fjx1, Fsh, Gamma Tubulin, Gna11, Grk6, Hsd17b1, Klf11, Lh, Lmcd1, Mapk1, Mapk1ip1, Mapkapk2, Mcl1, Mef2, Mef2b, Mlc, Mlcp, Myh4, Mylk2, Myosin-Light-Chain Kinase, Neurod4, Ngf, Notch, Nrg (Family), Oxsr1, Pawr, Pi3k (Family), Pkn1, Plat, Ppp6r1, Ptpase, Ptpra, Pyy, Rasal2, Rnf130, Secreted Mmp, Slx1a/Slx1b, Smad6, Snx18, Sod, Spata5l1, Src (Family), Stk39, Tcaf2, Timp2, Tm4sf1, Tmem158, Tmsb4, Tsc22d2

Disease Models & Mechanisms • Supplementary information

**6**	– Energy Production<br>– Lipid Metabolism<br>– Small Molecule Biochemistry	49	41	Adh1c, Agfg1, Akirin2, Akr1c3, Ap5z1, Bag1, Brms1l, C/Ebp, Calc, Cbp/P300, Cd207, Coup-Tf, Crebzf, Cuzd1, Cyp11b2, Dgk, Dgkz, Emcn, Erk1/2, Estrogen Receptor, Gc-Gcr Dimer, Gm9573, Hdac7, Histone Deacetylase, Histone H4, Hnf3, Hnf4g, Iga, Jink1/2, Jun/Junb/Jund, Klf9, Mettl16, Mknk2, Mrrf, Muc5b, Mucin, N-Cor, Ncor1, Nr0b2, Nr1h, Nr2f6, Pepck, Phf23, Pki, Pnrc1, Polr2m, Ppp1r3a, Rar, Rdh, Reg3g, Rxr, Sbsn, Sdr16c5, Sema3f, Serpinf1, Slc44a1, Spink5, Suds3, Swi-Snf, T3-Tr-Rxr, Tbx1, Tfiia, Thymidine Kinase, Thyroid Hormone Receptor, Tlr7/8, Tph2, Ube2j2, Ucp3, Vitamind3-Vdr-Rxr, Wdr92
**7**	– Connective Tissue Disorders<br>– Inflammatory Disease<br>– Organismal Injury and Abnormalities	44	38	Abhd17b, Ago2, Ago2-Mirlet7, Ankrd10, Ap1ar, Arl5a, Atp1b4, Cnot6, Cpd, Ddx56, Eif2, Eif2s1, Elavl1, Fam135a, Fam83f, Fbxo27, Gpr180, Gtpase, Hdhd2, Hras, Hsp90, Ifi27, Iigp1, Interferon Alpha, Ints11, Lamp1, Lmf1, Mex3d, Mir-323-3p (And Other Mirnas W/Seed Acauuac), Mir-330, Mir-363, Mir-423, Mir-423-5p (And Other Mirnas W/Seed Gaggggc), Mob3b, Nadk2, Nfic, Oaf, Oas1b, Pank1, Pank3, Pdcd7, Pdia4, Pp1/Pp2a, Ppfia1, Ppfibp2, Ppp2ca, Ppp2r3d, Pptc7, Proinsulin, Prom2, Rasa4, Rbpj, Rna Polymerase Ii, Rnase A, Sfta2, Sh3d19, Smad2/3, Smco3, Spast, Tarbp2, Tbc1d20, Tbc1d30, Tmem14a, Tmem65, Tnf (Family), Txnip, Ubp1, Ulk2, Xxylt1, Zcchc7
**8**	– Cell Death and Survival<br>– Cellular Compromise<br>– DNA Replication, Recombination, and Repair	40	36	7s Ngf, Alpha 1 Antitrypsin, Alpha Tubulin, Beta Tubulin, Ces, Ces1b/Ces1c, Collagen Type Ii, Ctla2a/Ctla2b, Cytokine Receptor, Ecm, Enac, Fam171a2, Fbxo31, Fbxw8, Fc Gamma Receptor, Fcer1, Fgd1, Granzyme, Gzma, Gzmb, Gzmc, Gzmh, Hsp, Ifit1, Ifn, Ifn Alpha/Beta, Ifn Beta, Ifn Type 1, Ifnar, Il12 (Complex), Il1r1, Il20rb, Il6, Jak, Kallikrein, Klk3, Klk7, Klrb1, Lilrb3, Map6, Mapkapk2/3, Mbp, Mep1a, Mhc, Mhc Class I (Complex), Mhc Class Ii (Complex), Mir122a, B, Mir124, Mirlet7, Msln, Mus81, Pdk2, Polg2, Prss3, Rgmb, Rnasel, Scarb2, Serine Protease, Serpina1, Serpina9, Syk/Zap, Tap2, Tec/Btk/Itk/Txk/Bmx, Th2 Cytokine, Tlr, Tmem189, Tmprss7, Trypsin, Txk, Vbp1
**9**	– Nervous System Development and Function<br>– Organ Morphology<br>– Tissue Morphology	35	33	Adam19, Adamtsl3, Agfg2, App, Arl5c, Arl8a, Arnt2, Brwd1, Cd200r1l, Cpn2, Csnk1g1, Ddx43, Dnajb14, Dnajc30, Entpd2, Fbxl20, Fcho1, Filip1, Gjd4, Gucd1, Gypa, H2-K2/H2-Q9, H2-M2, Hspb7, Il20rb, Il4, Jtb, Kcnq1, Kctd16, Krt40, Laptm4a, Lnx1, Ly6a (Includes Others), Ly6d, Mobp, Mturn, Myot, Nat3, Nudt11, Pcyt1b, Pdxp, Pkib, Purg, Rnf144b, Rnf38, Rwdd2b, Serpinb6b, Slc25a38, St6galnac6, Stac2, Tcp10/Tcp10l2, Tmem176b, Tmem59, Tp73, Traf6, Tshz2, Ubc, Ubl3, Ulk2, Usp6, Wars2, Wfdc8, Zadh2, Zdhhc14, Zfand5, Znf202, Znf706, Znrf1, Zscan16, Zxdc
**10**	– Amino Acid Metabolism<br>– Cardiovascular Disease<br>– Organismal Injury and Abnormalities	34	32	Actin, Agpat5, Alpha Actinin, Alpha Catenin, Angel2, Angpt2, Calpain, Camp-Dependent Protein Kinase, Casein, Chordc1, Cnga2, Collagen, Collagen Type I (Complex), Collagen Type I (Family), Collagen Type Iv, Collagen(S), Creb, Eif4ebp2, Eif4g, Eva1c, Exoc3, Exoc8, Fam136a, Fgfr, Focal Adhesion Kinase, G Protein Alphai, Gaba-A Receptor, Gabrg2, Gclc, Gclm, Gp6, Gria2, Growth Hormone, Gαq, Histone H1, Hrg, Hsp27, Importin Beta, Integrin, Itpka, Jak1/2, Laminin (Complex), Lfa-1, Lrp, Lum, Mapk, Mmp, Nup35, Pi3k (Complex), Pkc(S), Plc Beta, Plc Gamma, Pom121/Pom121c, Pp2a, Prap1, Prkcg, Ptk, Rab5, Secretase Gamma, Si, Spp1, Tars2, Tgf Beta, Ubqln1, Uros, Vps50, Vps51, Ythdc1, Ythdf1, Znf804a

E

E 7.25 6N Alcohol vs. Vehicle

	Diseases and Functions	-log(Fisher's exact *p*-value)	Molecules in Network	Molecules
**1**	– Cancer<br>– Gastrointestinal Disease<br>– Organismal Injury and Abnormalities	94	60	26s Proteasome, Ankrd13a, Apc (Complex), Arrdc1, Aven, Azin1, Bmt2, Btbd1, C1ql4, Caap1, Cab39, Cab39l, Carnmt1, Casein, Ccar1, Ccdc149, Cd200r1, Cdyl, Ctcfl, Cxxc1, Exog, Gmcl1, Haus4, Histone, Hivep3, Hoxc10, Kdm4b, Krt75, Leng8, Lypd3, Map4k2, Mark3, Mex3c, Mfhas1, Micu2, Mif4gd, Nfkb (Complex), Nr2e1, Parp, Parp16, Pdk2, Pip4k2a, Pip4k2b, Pkhd1, Plcd3, Prickle3, Prkaa, Pxk, Rbm26, Rcbtb2, Rnf25, Serpina12, Smurf1, Sult2b1, Tbrg4, Traf, Trib3, Trim13, Trim44, Trim69, Trmt1l, Ube2, Ube2g2, Ube2q2, Ubiquitin, Utp4, Znf263, Znf414, Znf761, Zranb1
**2**	– Cell Cycle<br>– Cellular Assembly and Organization<br>– DNA Replication, Recombination, and Repair	94	60	Actr5, Alpha Catenin, Anapc4, Atpase, Bbs4, Bcor, Cbp/P300, Ccdc117, Ccnl2, Cdc73, Cdipt, Ceacam, Ceacam20, Colec12, Dars1, Daxx, Fam161a, Fam91a1, Fbxo17, Fbxw21 (Includes Others), Figla, Fsd2, Histone H3, Hnrnph1, Hspa8, Igdcc3, Il1bos, Ilf2, Kdm1a, Klhl15, Lman2l, Mapk, Mapre1, Mettl3, Mi2, Myo19, Nlrp4b, Nsun4, Orc3, P-Tefb, Padi6, Pex6, Phkg2, Polr3h, Ppp2ca, Prkab1, Rbbp4, Rbpj, Rgs13, Rna Polymerase Ii, Rnr, Samd7, Smarca5, Sohlh1, Sp9, Spata19, Spice1, Spout1, Srsf2, Supt16h, Tcf19, Tmem25, Troap, Tssk1b, Tuba4a, Ythdc1, Ythdf1, Zbtb1, Znf639, Znf804a
**3**	– Gastrointestinal Disease<br>– Neurological Disease<br>– Organismal Injury and Abnormalities	59	44	Akr1c4, Amylase, Ankrd39, Anxa10, Arrb2, Bag3, Bdkrb1, Calmodulin, Card19, Cg, Chrm3, Ck2, Cpa3, Csnk1g3, Cyp4a11, Dazap2, Efcab5, Exoc3, Ffar2, Focal Adhesion Kinase, Fsh, Gbp6, Gnrh, Gpcr, Gpr160, Gpr4, Gpr50, Gpr88, Gtpase, Hemoglobin, Hsp90, Htr1d, Htr2b, Ice2, Ikk (Complex), Insulin, Klf1, Mtorc1, Ncbp2, Pka, Plc, Prmt3, Pstpip2, Ptprz1, Rab5c, Rac1, Ras Homolog, Rnf208, Rxfp1, Secretase Gamma, Sfk, Shc, Slc8b1, Slitrk1, Smarcal1, Src (Family), Sstr1, Stat, Stxbp4, Tas1r2, Tcf, Tcr, Tmem17, Tnk1, Tpcn2, Tsc22d1, Tsc22d2, Tubulin, Vamp2, Vegf

Disease Models & Mechanisms • Supplementary information

4	– Cell Morphology<br>– Cellular Development<br>– Cellular Movement	49	39	Acac, Actin, Afmid, Akirin2, Alp, Arp2/3, Arrdc4, Bcar1, Collagen, Collagen Alpha1, Collagen Type I (Complex), Collagen Type I (Family), Collagen Type Iv, Collagen(S), Commd3-Bmi1, Crebzf, Cuzd1, Cxcl13, Dynll2, Ecm, Erk1/2, Fam102a, Fap, Fcer1, Fermt1, Fgf, Fgf13, Fgf16, Fgf6, Fgf7, Fgfr, Gas1, Gp6, Gpiib-Iiia, Hcrt, Hedgehog, Hhat, Histone Deacetylase, Igsf3, Igsf8, Integrin, Itga3, Jink1/2, Laminin (Complex), Lfa-1, Loxl1, Lrig1, Mep1a, Myoc, Plc Gamma, Ppp1r15a, Rab5, Rap1, Rasgrp3, Rsk, Sh2d3c, Sharpin, Skap1, Slfn12l, Sox15, Srsf5, Talin, Tgf Beta, Tlr7/8, Ubiquitin Ligase, Ucn2, Wasf2, Wnt, Wnt8b, Zdhhc2
5	– Cell Cycle<br>– Developmental Disorder<br>– Endocrine System Disorders	47	38	Adaptor Protein, Adaptor Protein 2, Ap1s2, Ap2, Ap2 Alpha, Atn1, Beta Arrestin, Bmp15, Bok, Brms1l, Carboxylic Ester Hydrolase, Caspase, Caspase 3/7, Ccne2, Cdc2, Cdk, Cebpg, Ces, Ces1b/Ces1c, Ces1e, Ces4a, Cidec, Clathrin, Cpt1, Cyclin A, Cyclin D, Cyclin E, Dio1, Dynamin, Dzip3, E2f, E2f1, Hdac, Histone H4, Jnk, Kcnab3, Map1lc3, Mxd1, N-Cor, Ncor1, Nr1h, Pctp, Pepck, Polr2m, Ptgds, Rar, Rb, Rhobtb3, Rnf165, Rprd1a, Rxr, Shisa5, Siah2, Slc29a2, Smad1/5/9, Smad2/3, Ston2, Swi-Snf, Tars2, Tbx1, Tespa1, Tfap2b, Thymidine Kinase, Tnfrsf14, Uck2, Ulk2, Unc13d, Vps50, Wars2, Wdr62
6	– Hematological System Development and Function<br>– Lymphoid Tissue Structure and Development<br>– Tissue Morphology	45	37	Akt, Arfip2, C/Ebp, Cd180, Cd4, Chemokine, Chka, Cirbp, Cxcl16, Cytokine, Dsg1, Eif2ak2, Fcamr, Galnt1, Gm9573, Got, Grk6, Hk1, Ifn, Ifn Alpha/Beta, Ifn Beta, Ifnar, Iga, Ige, Igg, Igg1, Igg2a, Igg2b, Igg3, Igll1/Igll5, Igm, Ikb, Il1, Il12 (Complex), Il12 (Family), Il17d, Il36b, Immunoglobulin, Insrr, Interferon Alpha, Ly9, Mhc Class I (Complex), Mhc Class I (Family), Mhc Class Ii (Complex), Mhc Ii, Mlip, Mucin, Notch, Npas4, Pax5, Pdcd1lg2, Pro-Inflammatory Cytokine, Prr5, Rbm38, Reg3g, Saa3, Sema4b, Stat5a/B, Tlr, Tnf (Family), Tnfrsf17, Tnfsf12-Tnfsf13, Tnfsf13, Tnfsf15, Trem1, Trim59, Trpm4, Ugt2b17, Unc93b1, Vip
7	– Carbohydrate Metabolism<br>– Cell-To-Cell Signaling and Interaction<br>– Small Molecule Biochemistry	45	37	Abra, Adarb1, Adrb, Aldose Reductase, Alpha Tubulin, Ampa Receptor, Ampk, Ap1, Arf, Arhgap8/Prr5-Arhgap8, Bcr (Complex), C6, Calcineurin Protein(S), Camkii, Cd3, Clnk, Cofilin, Dock3, Epb41l1, Erk, Ern2, Flrt3, Gadd45a, Gcsam, Gk, Gria2, Histone H1, Ica1, Inpp5, Inpp5a, Inpp5k, Isl1, Kiaa1522, Lrrc10, Map2k1/2, Mgst2, Miip, Mlc, Mtch1, Mthfd2, Nck, Nck1, Nfat (Complex), P70 S6k, Pak, Pak6, Pde, Pdgf (Complex), Pdgf Bb, Pfkfb3, Pp1 Protein Complex Group, Pp2a, Proinsulin, Rac, Rhov, Rock, Sapk, Scarb2, Scd, Slc6a3, Sos, Srsf7, Ssbp4, Synj1, Tra2b, Trmt6, Tyrosine Kinase, Ubp1, Unc5b, Vav
8	– Energy Production<br>– Lipid Metabolism<br>– Small Molecule Biochemistry	42	35	14-3-3, Adcy, Adcy8, Alkbh3, Asb9, Atypical Protein Kinase C, Bbox1, Casp2, Creb, Cyct, Cyp2c8, Cytochrome C, Enac, Estrogen Receptor, F2rl1, G Protein, G Protein Alphai, G Protein Beta Gamma, Gnai1, Growth Hormone, Gsk3, Gαq, Hdl-Cholesterol, Helt, Hsp27, Hsp70, Irs1, Kcnj1, Klk3, Klra7 (Includes Others), Kndc1, Ldl, Ldl-Cholesterol, Lh, Mek, Muc5b, Neurog2, Nfat (Family), Nqo1, Nr0b2, Nr1d1, Nr2e3, Nr4a2, P38 Mapk, P85 (Pik3r), Paqr7, Pde3b, Pex14, Pi3k (Complex), Pi3k (Family), Pi3k P85, Pkc(S), Pnpla2, Prkd1, Prss29, Prss50, Ptpase, Rara, Ras, Serine Protease, Sod, T3-Tr-Rxr, Tmem54, Tmprss11b, Tmprss7, Trypsin, Tsh, Ucp2, Ucp3, Voltage-Gated Calcium Channel
9	– Cancer<br>– Cell Cycle<br>– Cellular Development	36	32	Acetyl-L-Carnitine, Acox, Acox1, Adss1, Ankar, Bco1, Bpifb6, C1orf159, Catsperg, Ces3, Cholesterol, Chtop, Dec1, Egr2, Fabp9, Fgf13, Fuca1, Fyn, Hcn2, Heparin, Hnrnpl, Hpd, Impg2, Inpp5a, Jpt2, Katnal1, Lrrc29, Lrtm1, Mir-100-5p (And Other Mirnas W/Seed Acccgua), Msl2, Mtmr11, Mup1 (Includes Others), Ncmap, Nfib, Nxph4, Obp2b, Ogdhl, Osbpl5, Pak6, Pctp, Pex2, Plekhg5, Pmm1, Pparg, Ppfia2, Ppp1ca, Ppp1r27, Ppp2r3d, Pstpip2, Ralgapa1, Rassf6, Rdh11, Rnf144b, Rorc, Shisa5, Shroom2, Slc16a14, Slc22a22, Slc22a25, Stard5, Tep1, Tex19.1, Tmem242, Tmem47, Tmem63a, Tmprss5, Tp53, Tpra1, Ubxn2b, Ywhaz
10	– Cell Cycle<br>– Cellular Assembly and Organization<br>– DNA Replication, Recombination, and Repair	35	31	Adal, Adamtsl3, Ammecr1, Amy2b, Arl5a, Asb15, Aspn, Aven, B4galnt3, C15orf39, Ccdc28a, Ccdc97, Cdc42se1, Cdh19, Cdr1, Ces2g, Cyp3a7, Ddx19a, Depdc7, Dmtn, Fam83g, Fermt1, Il10ra, Iws1, Kiaa0408, Klhdc3, Krtap10-3, Meikin, Meox2, Mex3d, Mobp, N4bp3, Npm1, Nr3c1, Nxf1, P3h2, Pax1, Paxbp1, Pnoc, Rad21, Rad21l1, Rdh11, Rec8, Reg4, Rgs18, Rnf144b, Rpp25, Rpp40, Sectm1, Selenop, Slc16a11, Slc22a16, Stag3, Sycp3, Tbccd1, Tcf3, Terb1, Tgfb1, Tlr7, Tmem104, Tomm34, Tomm40l, Tpst2, Tsen54, Ubl4b, Vgll2, Vim, Xpo1, Znf544, Znf780a

F

E 7.5 6J Alcohol vs. Vehicle

	Diseases and Functions	-log(Fisher’s exact *p*-value)	Molecules in Network	Molecules
1	– Dermatological Diseases and Conditions<br>– Lipid Metabolism<br>– Organismal Injury and Abnormalities	75	70	A4galt, Abhd17b, Agtrap, Arl14ep, Aunip, Carmil2, Casq1, Casq2, Ccdc184, Ccdc89, Ctnnbip1, Daglb, Dolpp1, Eaf1, Ensa, Epb41l5, Eri2, Extl1, Faf2, Fam114a1, Fam234a, Fbxo28, Gbx2, Gdf5, Gon7, Gpank1, Grb2, Grina, Hcn2, Hip1r, Ick, Inca1, Jsrp1, Kbtbd2, Klhdc3, Klhl36, Klk14, Lrrc8a, Lrrc8e, Lyg2, Map3k6, Ntf4, Pard6g, Pkp3, Plcd1, Plcd3, Plekho2, Pnma2, Pnma8a, Pop7, Ppp2r2d, Prap1, Prickle3, Rp1l1, Selenbp1, Snapc1, Snapc3, Spata2l, Stambpl1, Syt16, Tmem102, Ttyh2, Tulp1, Ubxn2a, Ubxn2b, Ubxn7, Ulk2, Vezt, Zfand2b, Zmym6

2	– Cell Death and Survival<br>– Cell Morphology<br>– Cellular Compromise	75	70	Adat1, Anln, Anxa6, Anxa7, Atxn7l3b, C18orf54, C19orf44, Canx, Casd1, Ccdc127, Cdv3, Cipc, Dctd, Def6, Dpep3, Dynll1, Eed, Elavl1, Eml2, Fam76a, Fam83f, Gipc1, Gk5, Gpm6b, Gsn, Gvin1 (Includes Others), Hm13, Hpdl, Inppl1, Kcng3, Lgals3bp, Lmf1, Lrrc1, Lrtomt, Mfsd13a, Mkrn2os, Mob3a, Myo1c, Osgin2, Pafah1b1, Pcmtd1, Pink1, Plekhg6, Proca1, Prrc1, Pskh1, Pwp2, Rbmxl1, Rbmxl2, Rdh13, Sfn, Slc50a1, Slc66a2, Smim14, Surf6, Thap3, Tmem189, Tmem53, Tmem68, Trim14, Trim25, Ttc7a, Uros, Vim, Wdr41, Ywhah, Zbtb41, Zfx, Znf354b, Znf91
3	– Cancer<br>– Connective Tissue Disorders<br>– Organismal Injury and Abnormalities	72	69	Arl8a, Arpc1b, Cd101, Cenpb, Clrn1, Cmbl, Cntn2, Col11a1, Col4a5, Col8a1, Collagen, Ctsf, Dhrs7b, Dnlz, Errfi1, Esyt1, Fam13b, Fbxl2, Foxl1, Hells, Hydin, Jagn1, Kiaa0930, Klhl21, Klhl26, Me1, Metap1, Mllt10, Mxd3, Mxi1, Naa16, Naa40, Nars1, Ndufaf4, Nfxl1, Nol9, Nrp1, Pdrg1, Pef1, Pogk, Polr2m, Ppp1r16a, Puf60, Pxn, Rabggtb, Rbm22, Rcbtb2, Rfwd3, Ripk4, Rmnd5b, Rnf146, Rnf19b, Rpusd1, Samhd1, Sema6c, Smarcad1, Tmpo, Topors, Tram2, Trim31, Tspan11, Ube2d4, Ubox5, Ubtd1, Vwa1, Wars2, Ypel5, Zfp42, Zg16, Zmynd19
4	– Cancer<br>– Organismal Injury and Abnormalities<br>– RNA Post-Transcriptional Modification	67	67	Abi2, Adal, Ai987944 (Includes Others), Atg16l1, Atp11b, Bricd5, Cbx1, Cbx5, Cebpa, Coil, Cxxc1, Dazap1, Dedd2, Dhx15, Efhd2, Fgf17, Grwd1, Hebp2, Histone H3, Hnrnpk, Hoxa9, Hp1bp3, Igsf8, Ilf2, Itga4, Kank3, Krt7, Lmna, Lsm2, Lsm6, Magea3 (Includes Others), Maged2, Mmd2, Pcid2, Pou2f3, Ppp4r2, Prpf38b, Ptgr1, Ptrhd1, Rbbp5, Rbm18, Riox2, Rnpc3, Rpl27a, Shmt2, Slc35a4, Smndc1, Snrnp, Snrpd1, Syngr1, Tada1, Tcp11l1, Thoc1, Tmem14a, Tmem203, Tmem267, Tmem42, Tmem54, Tmem86a, Trappc13, Tuba4a, U1 Snrnp, Ubn1, Wsb1, Yipf6, Zfp1, Znf22, Znf280d, Znf623, Znf688
5	– Cellular Assembly and Organization<br>– Cellular Function and Maintenance<br>– Infectious Diseases	65	66	Alg2, Ano10, Arfgap2, Arrdc1, Atf1, Bc048679, Cdc42ep2, Cenpn, Crim1, Dbr1, Escrt1, Esr1, Fam118a, Fam136a, Fdxacb1, Fibp, Fkbp1b, Gatb, Gc-Gcr Dimer, Gjd3, Hacd1, Hnf4g, Ica1, Kcnq4, Kctd17, Lor, Micu3, Mier1, Miip, Mkks, Mob3c, Ncoa7, Npw, Nsd1, Nup35, Paqr4, Pcdhb14, Pcdhb4, Peptidylprolyl Isomerase, Plscr3, Plxdc1, Pnrc1, Pnrc2, Ppwd1, Prr15l, Pus1, Qrsl1, Rar, Rarg, Rnf138, Rnf38, Sec22c, Shroom1, Slc44a2, Slco1c1, Stk16, Tfap2c, Them6, Tjp3, Trim47, Tsg101, Ttc9, Vps37a, Vps37b, Vps37d, Wdr4, Xpnpep1, Ypel3, Zdhhc21, Zfand4
6	– Embryonic Development<br>– Organ Development<br>– Organismal Development	65	66	Actc1, Actr1a, Aipl1, Ankrd34c, Atf7, Atpase, Borcs6, Borcs8, Ccdc8, Dctn2, Dtnbp1, Eif3j, Entpd1, Fbxo25, Fgfr1op, Foxc1, Gemin2, Gins1, Haus8, Hnrnph1, Jdp2, Kcnab3, Kiaa0753, Kiz, Krt16, Kti12, Mab21l3, Mb21d2, Med4, Mettl3, Mphosph9, Myh14, Myo18b, N4bp3, Ncbp2, Nlrp10, Nop2, Odf2l, P Glycoprotein, Panx1, Par, Patz1, Pfkp, Phax, Prickle2, Psmc5, Rhof, Sec31b, Shtn1, Snw1, Sorbs1, Spats2, Spdl1, Swsap1, Tcf15, Tcp1, Tcp11l2, Thg1l, Tnni3k, Tnnt2, Tor1aip1, Tor1b, Trap/Media, Txndc9, Usp9x, Vill, Wtap, Yap1, Znf326, Znf524
7	– Developmental Disorder<br>– Hereditary Disorder<br>– Post-Translational Modification	63	65	Amigo1, Asl, Atp8b2, Azin2, B3gnt3, B4galnt1, B4gat1, Btbd10, Btd, C1ql1, Ccdc106, Cd160, Cers4, Chodl, Cntnap3, Cryl1, Dtwd1, Ethe1, Evx1, Ext1, Fbxo2, Fev, Fktn, Fos, Galnt12, Gpha2, Gpt2, Has, Hus1, Interleukin, Kiaa1841, Klrg2, Lamb2, Lonrf2, Lrif1, Mad2l2, Metrn, Mob4, Mto1, Nagpa, Nkiras2, Ornithine Decarboxylase, Pcdh19, Pdf, Ppp2c, Prrx1, Ptcd2, Pxk, Rmnd1, Rtn2, Scf, Sgtb, Sike1, Slc10a6, Slc25a20, Smg7, Smg8, Snai3, Snx25, St6galnac4, St8sia3, St8sia5, Stk25, Strn3, Tbpl1, Tmem143, Tmem30b, Tpgs1, Xylt1, Znf146
8	– Cell Morphology<br>– Post-Translational Modification<br>– Small Molecule Biochemistry	63	65	Agfg2, Angel1, Aplp1, Atic, Atxn7l2, C1orf35, Ccdc122, Cdyl2, Chst13, Clock, Col9a2, Cspp1, Dennd2d, Dis3l, Epc1, Epc2, Eps15, Etv2, Exosc6, Exosc7, Exosome, Fez1, Fgf16, Foxn2, Foxo6, G2e3, Gcdh, Gnl1, Hap1, Hid1, Hmgcs2, Hoxb8, Hs3st3a1, Ing3, Ing5, Katnbl1, Meaf6, Med23, Mipol1, Mis12, Mphosph6, Nuf2, Papss2, Pdcd7, Pkdcc, Pkn3, Plvap, Pnp, Pxr Ligand-Pxr-Retinoic Acid-Rxrα, Rbm48, Rcn3, Reep3, Sec62, Sgf29, Slc25a41, Spc24, Specc1l, Srcap, Stx18, Sulfotransferase, Sult1d1, Sult1e1, Thap11, Tip60, Vegf, Vps51, Yeats4, Znf24, Znf324, Znf394
9	– Digestive System Development and Function<br>– Embryonic Development<br>– Post-Translational Modification	63	65	Abhd8, Ahcyl2, Ammecr1l, Anapc16, Arhgap45, Armc5, Banp, Bcap29, Bend3, Bmt2, Ccng1, Ccnl2, Cdx4, Cfap97, Chp1, Cog8, Dip2a, Fam117a, Fam124a, Fam161a, Fam214b, Farnesyl Transferase, Fnta, Gabp, Gabpb1, Gata, Gem, Ggtase I, Gmip, Hectd1, Hmbox1, Homer2, Iffo1, Irf2bp2, Irx5, Isl1, Itfg2, Lhx6, Lmo1, Lmo2, Lmo4, Loc728392, Lrfn4, Mbnl1, Meis2, Mnx1, Necap2, Nova1, Nudt22, Ooep, Pars2, Pggt1b, Pitx1, Proser2, Purg, Ras, Rundc3a, Ssbp4, Suox, Tal2, Tle6, Trim54, Uba6, Uck1, Vmac, Wdr33, Zbtb43, Zdhhc1, Znf513, Zzz3
10	– Cell Morphology<br>– Cellular Assembly and Organization<br>– Protein Synthesis	63	65	Adck5, Adss1, Appl2, Atm/Atr, Azin1, Chdh, Cpne7, Crlf3, Cxcl12, Dcaf15, Dtwd2, Eme2, Etaa1, Fa, Faap24, Fanc, Fancc, Fance, Gls, Grpel2, Grsf1, Gsap, Hoga1, Iba57, Id4, Lman2l, Mapre3, Mepce, Mfap3, Mgme1, Mmadhc, Mrm3, Mrn, Mrpl19, Mrpl21, Mrpl32, Mrpl39, Mrpl44, Mrpl45, Mrpl49, Mrpl9, Mrps25, Mrps27, Mrps30, Mrps7, Nit1, Oxld1, Pagr1, Psrc1, Rab31, Rmi2, Rpa, Rpusd3, Rpusd4, Sertad3, Slx1a/Slx1b, Slx4ip, Susd1, Tmc4, Tmco4, Tmem231, Tmem245, Top3a, Tra2a, Trmt1, Wfdc1, Xrcc2, Yars2, Ybey, Znf503

G — E 7.5 — 6N Alcohol vs. Vehicle

	Diseases and Functions	-log(Fisher's exact *p*-value)	Molecules in Network	Molecules

Disease Models & Mechanisms • Supplementary information

**1**	– Cell Morphology<br>– Embryonic Development<br>– Hair and Skin Development and Function	70	49	26s Proteasome, Adgrg6, Azin2, Calmodulin, Col4a6, Complement, Cxcr6, Dmc1, Dnajc6, Etv1, Fam222a, Fbn2, Fbxo6, Fpr1, Hemgn, Hnrnpk, Hspd1, Igg1, Igm, Il12 (Complex), Il12 (Family), Il1bos, Iqcb1, Kcnn1, Kcnn2, Keratin, Klhl22, Kpnb1, Krt16, Krt26, Krt33a, Krt35, Krt37, Krt73, Krt75, Krt86, Kyat1, Lgals3bp, Mapk, Mkrn3, Ndufa4l2, Noa1, Notch2, Notch3, Ornithine Decarboxylase, P-Tefb, Pbx1, Proinsulin, Pspc1, Ras, Rbm14, Rbmx, Rna Polymerase Ii, Sdr39u1, Sfpq, Sil1, Six2, Slc25a20, Speg, Sstr4, Stat, Syt2, Tbx19, Tcea2, Trim28, Trim31, Tuba4a, Tubulin, Ubr1, Znf462
**2**	– Cardiovascular Disease<br>– Cell Death and Survival<br>– Cellular Assembly and Organization	70	49	Adss1, Ak1, Ankrd37, Aven, Ccdc155, Ccnjl, Cga, Cldn13, Coq8b, Dio2, Dnpep, Egln, Fcn1, Fhl2, Gata2, Gmppa, Gpr158, Gpr25, Gpr37l1, Hebp2, Hmx1, Hnrnpl, Hnrnpu, Htr3b, Igsf9, Krtcap3, Kyat3, Lhfpl5, Lrrc57, Lyg2, Maml2, Marchf2, Mkrn2os, Morn1, Mtnr1b, Nanos2, Nr3c1, Nsg1, Nudt13, Odf4, Pdzph1, Pitx2, Prss56, Ptgr1, Retnlg, Rhag, Rhce/Rhd, Rhox3a (Includes Others), Rnf122, Rtn4rl1, Scube1, Selenbp1, Serp2, Serpinb9f (Includes Others), Serpini2, Slc44a4, Smim14, Sox13, Spire2, Stag3, Stfa2/Stfa2l1, Syngr1, Terb1, Tgfb1, Trim25, Trim58, Ubc, Upk3b, Wdpcp, Wdr34
**3**	– Lipid Metabolism<br>– Nucleic Acid Metabolism<br>– Small Molecule Biochemistry	58	43	Acss2, Acvr2b, Alp, Alpl, Ampk, Bcl11a, Bmp15, Bspry, Calcineurin Protein(S), Caspase 3/7, Cbp/P300, Ccnd2, Collagen Alpha1, Ctbp, Cuedc2, Cyclin A, Cyp11a1, Cyp2c40 (Includes Others), Cyp2c8, Cyp2e1, Cyp4f11, Dkk1, Elovl6, Emid1, Etv6, Fasn, Frizzled, Fsh, Gzmh, Hdac, Histone H3, Histone H4, Hsp70, Jnk, Kat2b, Khk, Lmcd1, Mef2, Mef2c, Mgmt, Mlycd, N-Cor, Nadh2 Or Nadph2 1 Atom Incorporation:Oxygen Oxidoreductase, Ncs1, Nr1h, P70 S6k, Prc2, Prkag2, Proc, Qki, Rab29, Rb, Rbm15, Sat2, Scd2, Six3, Smad, Smad2/3, Sp7, Tcf, Tgf Beta, Tgm2, Tnfrsf14, Tnn, Tp63, Trerf1, Unspecific Monooxygenase, Vgll2, Vill, Wnt1
**4**	– Cellular Movement<br>– Hematological System Development and Function<br>– Post-Translational Modification	58	43	2210010c04rik, Abl1, Adam22, Alpha 1 Antitrypsin, Ap1, Arrdc1, Atp6v1g3, Blvrb, Calpain, Caspase, Cd44, Cilp, Ck2, Cog8, Crabp2, Cstf3, Ctla2a/Ctla2b, Cytochrome C, Cytokine, Dsg1, Ecm, Efna4, Entpd1, F9, Focal Adhesion Kinase, Gbe1, Gcdh, Gsk3, H2ax, Histone, Hnrnpa1, Hnrnpdl, Hsp90, Il1r2, Immunoglobulin, Interleukin, Klk12, Lancl2, Ldb1, Ldl, Macrod1, Mdn1, Metalloprotease, Micb, Mmp, Mmp2, Necap2, P Glycoprotein, P38 Mapk, Parp, Phosphatase, Pink1, Pkc(S), Plppr3, Prap1, Prickle3, Prkch, Prss50, Prss56, Serine Protease, Sgca, Shp, St3gal2, Stx4, Tet1, Trypsin, Ubiquitin, Usp17le (Includes Others), Utp20, Vegf

**5**	– Developmental Disorder<br>– Organismal Injury and Abnormalities<br>– Skeletal and Muscular Disorders	54	41	Alpha Actinin, Amylase, Anxa4, Bmp, C3, Camk1, Cd55, Cd68, Cdh11, Cer1, Chst3, Chymotrypsin, Col10a1, Collagen Type I (Complex), Collagen Type Ii, Collagen Type Iii, Collagen Type Iv, Collagen Type V, Collagen Type X, Collagen(S), Complement Component 1, Cpa1, Cr3, Elastase, Erk1/2, Etv2, Fbxl12, Fgf, Fgf13, Fibrin, Fibrinogen, Gli, Gli3, Glipr2, Hcrt, Hgh1, Hopx, Hs6st2, Inppl1, Integrin, Klf13, Klrc1, Laminin (Complex), Lrg1, Mmp8, Nkx2-5, Pdgf (Complex), Pla2, Pla2g5, Pld, Pld2, Prrx1, Pxr Ligand-Pxr-Retinoic Acid-Rxrα, Rab26, Rab3d, Rgs5, Rps6ka, Rsph6a, Serpinb7, Ski, Slco1b3, Smooth Muscle Actin, Sox17, St6galnac3, Sulfotransferase, Sult1e1, Tbx1, Ubash3a, Ucn3, Wnt
**6**	– Cellular Movement<br>– Hematological System Development and Function<br>– Immune Cell Trafficking	52	40	Ackr2, Acox, Acy1, Akt, Asb9, Atrial Natriuretic Peptide, Ccl1, Ccl22, Ccl24, Ccl7, Ccng1, Collagen Type I (Family), Cox6a2, Creatine Kinase, Creb3l3, Ctla4, Cxcl13, Cxcl3, Cyp7a1, Dbh, Dynamin, Fcer1, Fgd2, Foxp1, Gdf15, Guca1a, Hfe, Ige, Il-2r, Il17r, Il17rc, Il23, Il2rb, Jak, Jink1/2, Jun/Junb/Jund, Laminin1, Loc102724788/Prodh, Lpin1, Map2k1/2, Marco, Mcam, Mek, Mir122a, B, Nfatc4, Or51e2, Pi3k (Family), Pi3k P85, Pik3c2g, Plac8, Plk3, Ppp2c, Prcp, Raf, Rap1, Rar, Reg3a, Rsk, Rxr, Rxrg, S100, S100a4, S100a8, S100a9, Scgb3a1, Serpinb6b, Slc6a12, T3-Tr-Rxr, Thyroid Hormone Receptor, Vla-4
**7**	– Endocrine System Development and Function<br>– Inflammatory Response<br>– Organismal Injury and Abnormalities	52	40	2' 5' Oas, 9830107b12rik/A530064d06rik, Alb, Aldh, Apyrase, Aqp5, C/Ebp, Capg, Cd200r1, Cd209c, Cd276, Chemokine, Collagen, Cspg4, Cyp1a1, Cytokine Receptor, Elf3, Filamin, Fuca1, Gadd45b, Gngt1, Growth Hormone, Hat, Hdl, Hif1, Hp, Ifn, Ifn Beta, Ifna4, Ifnar, Il1, Il36g, Il36rn, Interferon Alpha, Krtap11-1, Lcn2, Ldl-Cholesterol, Mapk13, Mhc Class Ii (Complex), Mus81, Nap1l2, Nfkb (Complex), Nlr, Nlrp4, Nlrp6, Notch, Oas, Oas1, Oas1b, Oas1d (Includes Others), Pepck, Polr3gl, Pro-Inflammatory Cytokine, Prtn3, Rras, Sapk, Setbp1, Slc46a2, Ssbp4, Stap2, Tgfbr, Tlr, Tnf (Family), Tnfrsf17, Tnfsf8, Ugt, Ugt2a1, Ugt2b17, Unc93b1, Vcan
**8**	– Cancer<br>– Organismal Injury and Abnormalities<br>– Psychological Disorders	50	39	Adcy, Adrb, Akr1b7, Anxa9, Ap2, Bace1, Bcas1, Bco1, Beta Arrestin, Blk, Cadm4, Cg, Creb, Estrogen Receptor, Fam3d, Fhl5, Flot1, Foxo6, G Protein Alphai, Gabrg2, Gimap8, Gipc1, Gnao1, Gnat3, Got, Gpcr, Hnrnph1, Htr1a, Igf2bp3, Insulin, Kcnj1, Kdr, Kiss1r, Ldh (Complex), Lh, Mediator, Nefm, Neurod2, Ntrk3, P85 (Pik3r), Pcyt1b, Pdgfr, Pi3k (Complex), Pka, Pka Catalytic Subunit, Plc, Pp2a, Ppm1j, Ppy, Ptpase, Ptpn1, Ptpn18, Rassf2, Ros1, Sfk, Shc, Sod, Src (Family), Srxn1, Stat5a/B, Trh, Trk Receptor, Tro, Tsh, Tspo, Tubb4a, Tubulin (Family), Voltage-Gated Calcium Channel, Ypel3, Znf326

Disease Models & Mechanisms • Supplementary information

**9**	– Cellular Function and Maintenance<br>– Cellular Growth and Proliferation<br>– Hematological System Development and Function	43	35	Acr, Actin, Alpha Catenin, Alpha Tubulin, Arhgap9, Arhgef2, Bach2, Bcr (Complex), Camkii, Cap2, Cblc, Cd207, Cd3, Cofilin, Ctnna3, Eps8l2, Erk, Erm, F Actin, Frs3, Gcsam, Gm9573, Gmpr, Grik5, Gsn, H2-L, H2-M10.1 (Includes Others), Hla-A, Hla-G, Iga, Igg, Igg2a, Igg3, Iqub, Itgad, Jph4, Kcna5, Klra7 (Includes Others), Mapk15, Mhc, Mhc Class I (Complex), Mhc Class I (Family), Mlc, Mucin, Myosin, Nadph Oxidase, Nfat (Complex), Nfat (Family), Pak, Pdgf Bb, Plc Gamma, Ptk, Rab11, Rac, Rac3, Ras Homolog, Rhoc, Rnd2, Rock, Sec14l1, Serpinb10, Sos, Sptbn4, St8sia3, Tap, Tapbp, Tcr, Tesk1, Trio, Tyrosine Kinase
**10**	– Cell Death and Survival<br>– DNA Replication, Recombination, and Repair<br>– Nervous System Development and Function	41	34	Acss2, Acy3, Adra2b, Aoc1, Apoc3, Asl, Bmper, Bpifb2, C11orf71, Calcrl, Ccer1, Ccr5, Ces2e, Cst9l, Cxcr6, Cyp2a12/Cyp2a22, Depp1, Dhx29, Dmc1, Fgf13, Fxyd6, Fxyd7, Fzd3, Fzd6, Gchfr, Gdpd4, Gpr37, Gpr37l1, Hdl-Cholesterol, Hipk1, Hnf4a, Ikzf2, Il10ra, Impg1, Kcnk3, Krt8, Loc102724788/Prodh, Map3k8, Mutyh, Mycn, Ndrg4, Npffr2, Npm1, Npnt, Nrarp, Nynrin, Plscr1, Prkcz, Rhox6/Rhox9, Rnase4, Sdr42e1, Sh3bgrl2, Slc10a5, Slc39a2, Sox2, Speer4a (Includes Others), Sprr2h, Sprr2i, Sstr4, Sycn, Tada1, Tapbpl, Tet1, Tex15, Tm6sf1, Tp53, Unc5a, Urah, Yy2, Zic3

Disease Models & Mechanisms • Supplementary information

Table S3. Selected pathways significantly enriched among differentially expressed genes between the vehicle-treated 6J and 6N embryos 6 hr after injection (E7.25).

Down-regulated Genes

Gene Ontology: Biological Process

Term name	Term ID	# of genes	Log10 *p*-value
Cytolysis	GO:0019835	7	5.12
Granzyme-mediated apoptotic signaling	GO:0008626	5	4.85

Gene Ontology: Molecular Function

Term name	Term ID	# of genes	Log10 *p*-value
Serine-type endopeptidase activity	GO:0004252	11	4.30
Hydrolase activity, acting on acid phosphorus-nitrogen bonds	GO:0016825	11	3.80
Serine hydrolase activity	GO:0017171	11	3.80
Peptidase activity	GO:0008233	16	2.18
Peptidase activity, acting on L-amino acid peptides	GO:0070011	15	1.79

KEGG: Biological Process

Term name	Term ID	# of genes	Log10 *p*-value
Graft-versus-host disease	KEGG:05332	5	2.71
cAMP signaling	KEGG:04024	9	2.68
Neuroactive ligand-receptor interaction	KEGG:04080	10	1.84

Reactome

Term name	Term ID	# of genes	Log10 *p*-value
Activation, myristolyation of BID and translocation to mitochondria	REAC:R-MMU-75108	5	5.59
Intrinsic pathway for apoptosis	REAC:R-MMU-109606	5	2.63

Up-regulated Genes

KEGG: Biological Process

Term name	Term ID	# of genes	Log10 *p*-value
Caffeine metabolism	KEGG:00232	2	1.94

Disease Models & Mechanisms • Supplementary information

Table S4. Selected pathways significantly enriched among differentially expressed genes between the 6J and 6N embryos 12 hr after vehicle treatment (E7.5).

Down-regulated Genes

Gene Ontology: Molecular Function

Term name	Term ID	# of genes	Log10 *p*-value
Serine-type endopeptidase activity	GO:0004252	9	2.62
Serine-type peptidase activity	GO:0008236	9	2.30
Hydrolase activity, acting on acid phosphorus-nitrogen bonds	GO:0016825	9	2.22
Serine hydrolase activity	GO:0017171	9	2.22
Endopeptidase activity	GO:0004175	12	1.60

Up-regulated Genes

Reactome

Term name	Term ID	# of genes	Log10 *p*-value
Peptide ligand-binding receptors	R-MMU-375276	7	2.74
GPCR ligand binding	R-MMU-500792	9	2.58
Class A/1 (Rhodopsin-like receptors)	R-MMU-373076	8	2.42

Human Phenotype Ontology

Term name	Term ID	# of genes	Log10 *p*-value
Hyperprostaglandinuria	HP:0003527	2	1.93
Increased serum prostaglandin E2	HP:0003566	2	1.93
Renal juxtaglomerular cell hypertrophy/hyperplasia	HP:0000111	2	1.93
Abnormal circulating prostaglandin circulation	HP:0011023	2	1.93

Disease Models & Mechanisms • Supplementary information

Table S5. Selected pathways significantly enriched among differentially expressed genes between the PAE and vehicle-treated 6J embryos 6 hr after exposure (E7.25).

Down-regulated Genes

Gene Ontology: Biological Process

Term name	Term ID	# of genes	Log10 *p*-value
Cellular metabolic process	GO:0044237	275	10.45
Gene expression	GO:0010467	162	6.44
Methylation	GO:0032259	27	5.47
Protein modification process	GO:0036211	119	4.32
Cell cycle	GO:0007049	66	3.91
Cellular biosynthetic process	GO:0044249	154	2.96
RNA modification	GO:0009451	14	2.69
Chromosome organization	GO:0051276	45	1.73
Programmed cell death	GO:0012501	65	1.43
Transcription by RNA polymerase II	GO:0006366	66	1.39

Gene Ontology: Molecular Function

Term name	Term ID	# of genes	Log10 *p*-value
Heterocyclic compound binding	GO:1901363	172	7.10
Methyltransferase activity	GO:0008168	19	4.81
Catalytic activity	GO:0003824	159	3.24
Nucleic acid binding	GO:0003676	110	3.05
Enzyme binding	GO:0019899	76	2.66
ATP binding	GO:0005524	55	2.57
Protein binding	GO:0005515	241	2.51
miRNA binding	GO:0035198	6	1.83
Ribonucleotide binding	GO:0032553	64	1.82
transcription regulator activity	GO:0140110	49	1.31

Human Phenotype Ontonology

Term name	Term ID	# of genes	Log10 *p*-value
Broad forehead	HP:0000337	13	2.57

Disease Models & Mechanisms • Supplementary information

Up-regulated Genes

Gene Ontology: Biological Process

Term name	Term ID	# of genes	Log10 *p*-value
Cytolysis	GO:0019835	9	5.53
Granzyme-mediated apoptotic signaling	GO:0008626	5	3.19
Primary alcohol metabolic process	GO:0034308	8	1.69

Gene Ontology: Molecular Function

Term name	Term ID	# of genes	Log10 *p*-value
Serine-type endopeptidase activity	GO:0004252	12	2.14
Endopeptidase activity	GO:0004175	19	1.93
Hydrolase activity, acting on acid phosphorus-nitrogen bonds	GO:0016825	12	1.66
Serine hydrolase activity	GO:0017171	12	1.66

KEGG: Biological Process

Term name	Term ID	# of genes	Log10 *p*-value
Graft-versus-host disease	KEGG:05332	5	1.38

Reactome

Term name	Term ID	# of genes	Log10 *p*-value
Activation, myristolyation of BID and translocation to mitochondria	REAC:R-MMU-75108	5	4.08

Disease Models & Mechanisms • Supplementary information

Table S6. Selected pathways significantly enriched among differentially expressed genes between the PAE and vehicle-treated 6N embryos 6 hr after exposure (E7.25).

Down-regulated Genes

Gene Ontology: Biological Process

Term name	Term ID	# of genes	Log10 *p*-value
Organic substance metabolic process	GO:0071704	179	3.38
Macromolecule metabolic process	GO:0043170	159	3.01
Primary metabolic process	GO:0044238	171	2.98
Methylation	GO:0032259	18	2.71
Metabolic process	GO:0008152	182	2.27
Macromolecule methylation	GO:0043414	16	2.17
Regulation of metabolic process	GO:0019222	119	1.71
Nitrogen compound metabolic process	GO:0006807	159	1.68
RNA metabolic process	GO:0016070	87	1.67
Regulation of cellular metabolic process	GO:0031323	111	1.44

Gene Ontology: Molecular Function

Term name	Term ID	# of genes	Log10 *p*-value
rRNA (adenine) methyltransferase activity	GO:0016433	3	1.74
Catalytic activity	GO:0003824	107	1.62
Sterol esterase activity	GO:0004771	4	1.50
Methyltransferase activity	GO:0008168	11	1.35

Reactome

Term name	Term ID	# of genes	Log10 *p*-value
Gene expression (transcription)	R-MMU-74160	29	1.78
RNA polymerase II transcription	R-MMU-73857	27	1.73

Disease Models & Mechanisms • Supplementary information

Table S7. Selected pathways significantly enriched among differentially expressed genes between the PAE and vehicle-treated 6J embryos 12 hr after exposure (E7.5).

Down-regulated Genes

Gene Ontology: Biological Process

Term name	Term ID	# of genes	Log10 *p*-value
Nucleic acid metabolic process	GO:0090304	552	69.99
Gene expression	GO:0010467	559	57.57
Primary metabolic process	GO:0044238	819	45.95
Embryo development	GO:0009790	136	13.2
Heart development	GO:0007507	77	7.33
Skeletal system development	GO:0001501	66	6.40
Cellular response to DNA damage stimulus	GO:0006974	82	4.75
Regulation of cell cycle	GO:0051726	100	4.02
Circulatory system development	GO:0072359	108	3.84
Brain development	GO:0007420	76	3.40

Gene Ontology: Molecular Function

Term name	Term ID	# of genes	Log10 *p*-value
Nucleic acid binding	GO:0003676	432	54.31
Organic cyclic compound binding	GO:0097159	550	40.57
DNA binding	GO:0003677	266	33.72
RNA binding	GO:0003723	156	18.46
Transcription factor binding	GO:0008134	88	9.27
Catalytic activity, acting on RNA	GO:0140098	55	7.99
Histone acetyltransferase activity	GO:0004402	14	2.62
Protein binding	GO:0005515	636	1.86
miRNA binding	GO:0035198	9	1.68
Histone binding	GO:0042393	30	1.50

KEGG: Biological Process

Term name	Term ID	# of genes	Log10 *p*-value
Spliceosome	KEGG:03040	22	3.67
Signaling pathways regulating pluripotency of stem cells	KEGG:04550	21	2.67
RNA transport	KEGG:03013	22	2.06
Ribosome biogenesis in eukaryotes	KEGG:03008	13	1.64

Reactome

Disease Models & Mechanisms • Supplementary information

Term name	Term ID	# of genes	Log10 *p*-value
Gene expression (transcription)	R-MMU-74160	112	6.92
Metabolism of RNA	R-MMU-8953854	72	6.67
Processing of capped intron-containing pre-mRNA	R-MMU-72203	38	4.93
RNA polymerase II transcription	R-MMU-73857	94	3.84
mRNA splicing	R-MMU-72172	29	3.04
mRNA splicing - major pathway	R-MMU-72163	28	2.86
RNA polymerase II transcribes snRNA genes	R-MMU-6807505	15	2.34

Human Phenotype Ontology

Term name	Term ID	# of genes	Log10 *p*-value
Abnormal lip morphology	HP:0000159	81	3.41
Abnormal palate morphology	HP:0000174	84	3.07
Abnormality of the outer ear	HP:0000356	89	2.93
Holoprosencephaly	HP:0001360	15	2.39
Abnormal facial shape	HP:0001999	80	1.73
Anal atresia	HP:0002023	21	1.65
Atrioventricular canal defect	HP:0006695	11	1.53
Oral cleft	HP:0000202	53	1.42

Up-regulated Genes

Gene Ontology: Biological Process

Term name	Term ID	# of genes	Log10 *p*-value
Localization	GO:0051179	529	9.04
Transport	GO:0006810	411	7.64
Transmembrane transport	GO:0055085	160	4.75
Protein phosphorylation	GO:0006468	189	4.25
Intracellular signal transduction	GO:0035556	236	4.11
Cellular response to chemical stimulus	GO:0070887	272	3.12
Anatomical structure development	GO:0048856	466	2.18
Response to stress	GO:0006950	316	1.71
Cell death	GO:0008219	191	1.48
Positive regulation of cell differentiation	GO:0045597	109	1.42

Disease Models & Mechanisms • Supplementary information

Gene Ontology: Molecular Function

Term name	Term ID	# of genes	Log10 *p*-value
Protein binding	GO:0005515	821	9.85
TAP binding	GO:0046977	8	4.52
GTP binding	GO:0005525	54	3.42
Nucleotide binding	GO:0000166	204	2.82
Enzyme binding	GO:0019899	207	1.79
Beta-2-microglobulin binding	GO:0030881	6	1.78
Cytoskeletal protein binding	GO:0008092	98	1.57
GTPase activity	GO:0003924	40	1.48
Protein kinase binding	GO:0019901	76	1.38

Reactome

Term name	Term ID	# of genes	Log10 *p*-value
Metabolism	R-MMU-1430728	158	1.78
Ion channel transport	R-MMU-983712	27	1.68
Stimuli-sensing channels	R-MMU-2672351	18	1.35
Formation of xylulose-5-phosphate	R-MMU-5661270	4	1.31

Table S8. Selected pathways significantly enriched among differentially expressed genes between the PAE and vehicle-treated 6N embryos 12 hr after exposure (E7.5).

<u>Down-regulated Genes</u>

Gene Ontology: Biological Process

Term name	Term ID	# of genes	Log10 *p*-value
Positive regulation of transcription by RNA polymerase II	GO:0045944	37	5.02
Positive regulation of cellular metabolic process	GO:0031325	69	4.56
Transcription by RNA polymerase II	GO:0006366	47	3.27
Regulation of cell differentiation	GO:0045595	44	2.61
Cell fate commitment	GO:0045165	13	1.85
Skeletal system development	GO:0001501	18	1.72
Primary metabolic process	GO:0044238	140	1.53
Embryonic morphogenesis	GO:0048598	20	1.52
Generation of neurons	GO:0048699	36	1.47
Regulation of nervous system development	GO:0051960	27	1.46

Gene Ontology: Molecular Function

Term name	Term ID	# of genes	Log10 *p*-value
Sequence-specific DNA binding	GO:0043565	38	6.17
RNA polymerase II regulatory region sequence-specific DNA binding	GO:0000977	29	5.17
Transcription regulatory region DNA binding	GO:0044212	32	5.15
Cis-regulatory region binding	GO:0035326	25	4.87
Transcription regulator activity	GO:0140110	37	3.50
DNA-binding transcription activator activity	GO:0001216	17	2.77
DNA binding	GO:0003677	43	1.65

KEGG: Biological Process

Term name	Term ID	# of genes	Log10 *p*-value
Steroid hormone biosynthesis	KEGG:00140	6	1.77

Human Phenotype Ontology

Term name	Term ID	# of genes	Log10 *p*-value
Hypoplasia of the epiglottis	HP:0005349	3	1.31

Disease Models & Mechanisms • Supplementary information

Up-regulated Genes

Gene Ontology: Biological Process

Term name	Term ID	# of genes	Log10 *p*-value
Antigen processing and presentation of endogenous antigen	GO:0019883	10	6.37
Antigen processing and presentation via MHC class Ib	GO:0002475	9	5.31
Antigen processing and presentation of peptide antigen	GO:0048002	10	4.18
Defense response	GO:0006952	52	3.78
Response to stress	GO:0006950	91	3.16
Antigen processing and presentation of peptide antigen via MHC class I	GO:0002474	6	3.07
Antigen processing and presentation	GO:0019882	11	2.87
Inflammatory response	GO:0006954	27	1.90

Gene Ontology: Molecular Function

Term name	Term ID	# of genes	Log10 *p*-value
TAP binding	GO:0046977	6	5.78
Beta-2-microglobulin binding	GO:0030881	6	5.78
Peptide antigen binding	GO:0042605	6	4.06
Catalytic activity	GO:0003824	121	2.68
RAGE receptor binding	GO:0050786	4	2.64

KEGG: Biological Function

Term name	Term ID	# of genes	Log10 *p*-value
IL-17 signaling pathway	KEGG:04657	8	1.74

Reactome

Term name	Term ID	# of genes	Log10 *p*-value
ER-phagosome pathway	R-MMU-1236974	8	4.39
Neutrophil degranulation	R-MMU-6798695	25	3.47
Endosomal/vacuolar pathway	R-MMU-1236977	6	2.66
Antigen presentation: Folding, assembly and peptide loading of class I MHC	R-MMU-983170	7	2.57
Metal sequestration by antimicrobial proteins	R-MMU-6799990	3	2.23
Peptide ligand-binding receptors	R-MMU-375276	12	1.60

Disease Models & Mechanisms • Supplementary information

Human Phenotype Ontology

Term name	Term ID	# of genes	Log10 *p*-value
Crazy paving pattern on pulmonary HRCT	HP:0025391	5	3.27
Elevated carcinoembryonic antigen level	HP:0031029	5	3.05
Recurrent acute respiratory tract infection	HP:0011948	6	2.94
Acute infectious pneumonia	HP:0011949	5	2.48
Foam cells	HP:0003651	5	2.18
Respiratory failure requiring assisted ventilation	HP:0004887	5	1.92
Abnormal blood oxygen level	HP:0500165	5	1.80
Hypoxemia	HP:0012418	5	1.80
Abnormal blood gas level	HP:0012415	5	1.48
Abnormal pulmonary thoracic imaging finding	HP:0031983	5	1.48

Table S9. Cilia-related genes significantly different between the strains at baseline or within the strains after alcohol.

GD7.0		GD7.25				GD7.5			
6J's vs. 6N's		6J's		6N's		6J's		6N's	
Baseline		PAE vs. Vehicle		PAE vs. Vehicle		PAE vs. Vehicle		PAE vs. Vehicle	
Gene	Log2 FC	Gene	Log2 FC	Gene	Log2 FC	Gene	Log2 FC	Gene	Log2 FC
Fam65b	0.30	*Fam161a*	*0.86*	*Fam161a*	*0.98*	*Mchr1*	1.28	*Iqub*	0.95
Efcab7	-0.89	*Tbc1d30*	*0.75*	*Tex40*	*0.94*	*Fam161a*	1.17	*Tekt1*	0.82
		Hydin	*0.65*	*Hk1*	*0.81*	*Hydin*	0.88	*Tulp1*	0.80
		Map6	*0.61*	*Ak1*	*0.80*	*Rab29*	0.85	*Rab29*	0.68
		Bbs4	*0.61*	*Tuba4a*	*0.61*	*Efcab7*	0.81	*Tuba4a*	0.67
		Ptch1	*0.46*	*Bbs4*	*0.52*	*Tmem231*	0.81	*Wdpcp*	0.62
		Arl2bp	*0.38*	*Tmem17*	*0.43*	*Mxi1*	0.80	*Cep72*	0.49
		Ttll1	*0.28*	*Haus4*	*0.35*	*Tuba4a*	0.78	*Wdr34*	0.48
		Cnga2	*0.041*	*Pkhd1*	*0.34*	*Rilpl2*	0.74	*Pkd2l1*	0.33
		Myoc	*0.02*	*Tekt3*	*0.03*	*Ccno*	0.72	*Ropn1*	-0.06
		Ccr6	*-0.03*	*Ttll8*	*0.01*	*Tulp1*	0.71	*Iqcb1*	-0.35
		Mapre1	*-0.17*	*Myoc*	*-0.06*	*Hspb11*	0.68	*Rsg1*	-0.80
		Cct3	*-0.21*	*Cngb3*	*-0.08*	*Rilpl1*	0.68	*Gli3*	-0.83
		Exoc3	*-0.23*	*Mapre1*	*-0.19*	*Mok*	0.60		
		Dnaja1	*-0.30*	*Exoc3*	*-0.24*	*Kiz*	0.56		
		Nup35	*-0.44*	*Hspa8*	*-0.36*	*Kif3b*	0.52		
		Hspa8	*-0.45*	*Ralgapa1*	*-0.51*	*Mdm1*	0.51		
		Gna11	*-0.48*	*Trim59*	*-0.56*	*Snx10*	0.44		
		Pard3	*-0.58*	*Katnal1*	*-0.58*	*Pde6d*	0.44		
		Tapt1	*-0.59*	*Pex6*	*-0.74*	*Usp9x*	0.44		
		Gnb1	*-0.71*	*Wdr78*	*-0.78*	*Prkar2a*	0.43		
		Dyx1c1	*-0.72*			*Ick*	0.40		
		Atp2a2	*-1.02*			*Tubb2a*	0.34		
		Prkaca	*-1.19*			*Unc119*	0.33		
		Pex6	*-1.38*			*Rp1l1*	0.07		
						Dpcd	-0.35		
						Plk4	-0.39		
						Aurka	-0.42		
						Fgfr1op	-0.48		
						Tmem80	-0.51		
						Topors	-0.56		
						Smo	-0.59		
						Gli3	-0.59		
						Pafah1b1	-0.59		
						Pifo	-0.61		
						Dnajb13	-0.65		
						Cspp1	-0.66		
						Rttn	-0.66		
						Flcn	-0.67		
						Noto	-0.68		
						Pkd2	-0.69		
						Rab11b	-0.69		
						Morn3	-0.72		
						Cfap161	-0.77		
						Ptch1	-0.79		
						Spef2	-0.83		
						Tapt1	-0.90		
						Mkks	-1.03		
						Osr1	-1.43		

Dataset 1. Excel file containing VST-normalized values for all replicates, group means, Log2FC, and adjusted p-values for all differentially expressed genes from each comparison.

Click here to download Dateset1
